# Directing ricin-based immunotoxins with targeting affibodies and KDEL signal peptide to cancer cells effectively induces apoptosis and tumor suppression

**DOI:** 10.1186/s12951-022-01601-8

**Published:** 2022-08-23

**Authors:** Seong Guk Park, Heeyeon Kim, Heejin Jun, Sun Young Choi, Eunhee Kim, Sebyung Kang

**Affiliations:** 1 Department of Biological Sciences, Ulsan National Institute of Science and Technology (UNIST), Ulsan, Korea; 2 Department of Medicine, Graduate School, Korea University, Seoul, Korea

**Keywords:** Ricin, Recombinant immunotoxin, Affibody molecule, KDEL, Tumor suppression, Intracellular delivery, Drug delivery

## Abstract

The plant toxin ricin, especially its cytotoxic A chain (RTA), can be genetically engineered with targeting ligands to develop specific anti-cancer recombinant immunotoxins (RITs). Here, we used affibody molecules targeting two cancer biomarkers, the receptors HER2 and EGFR, along with the KDEL signal peptide to construct two cancer-specific ricin-based RITs, HER2Afb-RTA-KDEL and EGFRAfb-RTA-KDEL. The affibodies successfully provided target-specificity and subsequent receptor-mediated endocytosis and the KDEL signal peptide routed the RITs through the retrograde transport pathway, effectively delivering RTA to the cytosol as well as avoiding the alternate recycling pathway that typical cancer cells frequently have. The in vivo efficacy of RITs was enhanced by introducing the albumin binding domain (AlBD) to construct AlBD/HER2Afb/RTA-KDEL. Systemic administration of AlBD-containing RITs to tumor-bearing mice significantly suppressed tumor growth without any noticeable side-effects. Collectively, combining target-selective affibody molecules, a cytotoxic RTA, and an intracellularly designating peptide, we successfully developed cancer-specific and efficacious ricin-based RITs. This approach can be applied to develop novel protein-based “magic bullets” to effectively suppress tumors that are resistant to conventional anti-cancer drugs.

**Graphical Abstract:**

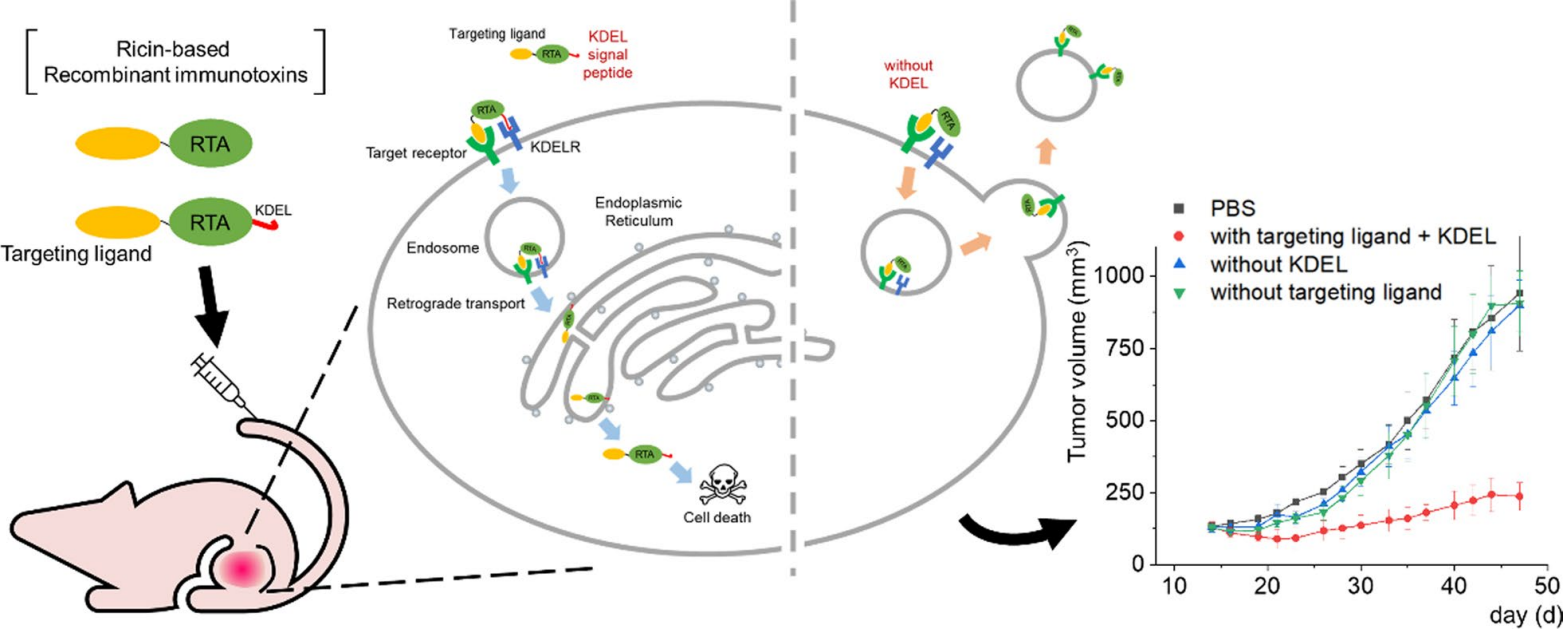

**Supplementary Information:**

The online version contains supplementary material available at 10.1186/s12951-022-01601-8.

## Background

Targeted delivery of anti-cancer drugs has been extensively studied to improve their therapeutic efficacy while minimizing side-effects and non-specific damages to normal cells and tissues. Various therapeutic agents, including small synthetic chemical drugs, radioisotopes, and therapeutic proteins, have often been conjugated to targeting ligands and selectively delivered to the tumor sites [1]. Among them, protein toxins are considered excellent candidates for developing target-specific anti-cancer drugs because they are highly potent and cytotoxic enzymes that have evolved to kill specific cells by enzymatically inactivating essential cellular processes [2]. Also, they can be engineered to incorporate targeting ligands and form recombinant immunotoxins (RITs), controlling cancer cells in a target-specific manner [1–4].

Ricin is a protein toxin produced by the seeds of the castor oil plant *Ricinus communis* [5]. It belongs to the AB toxin family, comprising A chain (RTA) and B chain (RTB) which are responsible for cytotoxic activity and target cell binding, respectively [6]. When the RTB of ricin holotoxin binds to a galactose moiety on the cell surface [7], the holotoxin is endocytosed. Subsequently, the RTB interacts with calreticulin, an endoplasmic reticulum (ER)-resident protein which retrogradely transports the bound holotoxin to the ER [8]. The RTA is cleaved from the holotoxin and delivered to the cytosol through the ER-associated protein degradation (ERAD) pathway [3, 9]. The RTA is a ribosome-inactivating protein and the delivered cytosolic RTA would hydrolyze adenine 4324 (A4324) in eukaryotic 28S rRNA, blocking host protein synthesis and eventually resulting in apoptotic cell death [10, 11].

By genetically replacing the native RTB of ricin holotoxin with cancer-targeting ligands, ricin-based RITs can become potential anti-cancer drugs. Various targeting ligands, including natural receptor-binding ligands [4], targeting peptide [12–14], monoclonal antibodies [15], antibody derivatives [15, 16], and antibody mimetics [17–19], have been used to develop new target-specific RITs. Antibody mimetics are molecules that are not related to antibodies but show high affinity and specificity towards particular molecules or antigens [20]. Affibody molecules are representative antibody mimetics derived from the Z domain of staphylococcal protein A [21]. They are small (~ 10 kDa), easily produced in *Escherichia coli* in large quantities, show high affinity and selectivity for their targets even when genetically fused with other proteins [21], act as separated domains, and do not require any further chemical conjugation. However, replacing the RTB with other targeting ligands often lowers the cytotoxicity of the RIT due to the loss of ER-targeting capability of ricin. Therefore, it is necessary to preserve the intracellular route of recombinant toxins. The KDEL (Lys-Asp-Glu-Leu) signal peptide could be useful in this regard because it is a well-known ER-targeting sequence [22, 23].

In this study, we constructed ricin-based RITs comprising the RTA, affibody molecules, and the KDEL signal peptide, and evaluated their target-specific cytotoxicity against corresponding target cancer cells. In addition, we monitored the intracellular localization of the RTA in the presence or absence of the KDEL signal peptide using a fluorescence microscope. Finally, we explored the translational implications of ricin-based RITs by examining their efficacy in mouse models of cancer.

## Results and discussion

### KDEL signal peptide in combination with targeting affibody molecules improves cytotoxicity of RTA against target cancer cells

Human epidermal growth factor receptor 2 (HER2) and epidermal growth factor receptor (EGFR; HER1) are representative cancer markers that are overexpressed on the surface of several cancer cells [24, 25]. Therefore, we used affibodies that target HER2- or EGFR-overexpressing cancer cells [21] while designing our RITs. The coding sequence of an affibody molecule that binds to HER2 (ZHER2:342; HER2Afb) or EGFR (ZEGFR:1907; EGFRAfb) [26, 27] was genetically fused to the N-terminus of RTA (residues 36–302) to obtain RTA-based RIT clones (Additional file 1: Fig. S2A and B). The constructed HER2Afb-RTA and EGFRAfb-RTA clones were overexpressed in E. coli BL21 (DE3) strain and purified by immobilized metal affinity chromatography (Additional file 1: Fig. S2C).

Next, we performed cell viability assays to quantitatively examine target-specific cytotoxicity of each RIT. We chose NIH3T6.7 (immortalized mouse fibroblast cell) and SK-BR-3 (human breast cancer cell) as HER2-overexpressing cancer cell lines, and A431 (human skin cancer cell) and MDA-MB-468 (human breast cancer cell) as EGFR-overexpressing cancer cell lines. We treated them with HER2Afb-RTA or EGFRAfb-RTA. However, cytotoxic effects of the RITs against their corresponding target cells were much lower than expected (Additional file 1: Fig. S1). While the HER2Afb-RTA showed higher cytotoxicity against NIH3T6.7 cells than doxorubicin did, EGFRAfb-RTA exhibited similar cytotoxicity as doxorubicin against MDA-MB-468 cells (Additional file 1: Fig. S1A and C). In addition, both the RITs showed almost no cytotoxicity against SK-BR-3 and A431 cells (Additional file 1: Fig. S1B and D). The RTB is known to not only bind to its receptor but also retrogradely transport the RTA to the ER [8] and replacement of the RTB with either HER2Afb or EGFRAfb may alter the destination of the RTA, resulting in the significant loss of cytotoxicity.

**Fig. 1:**
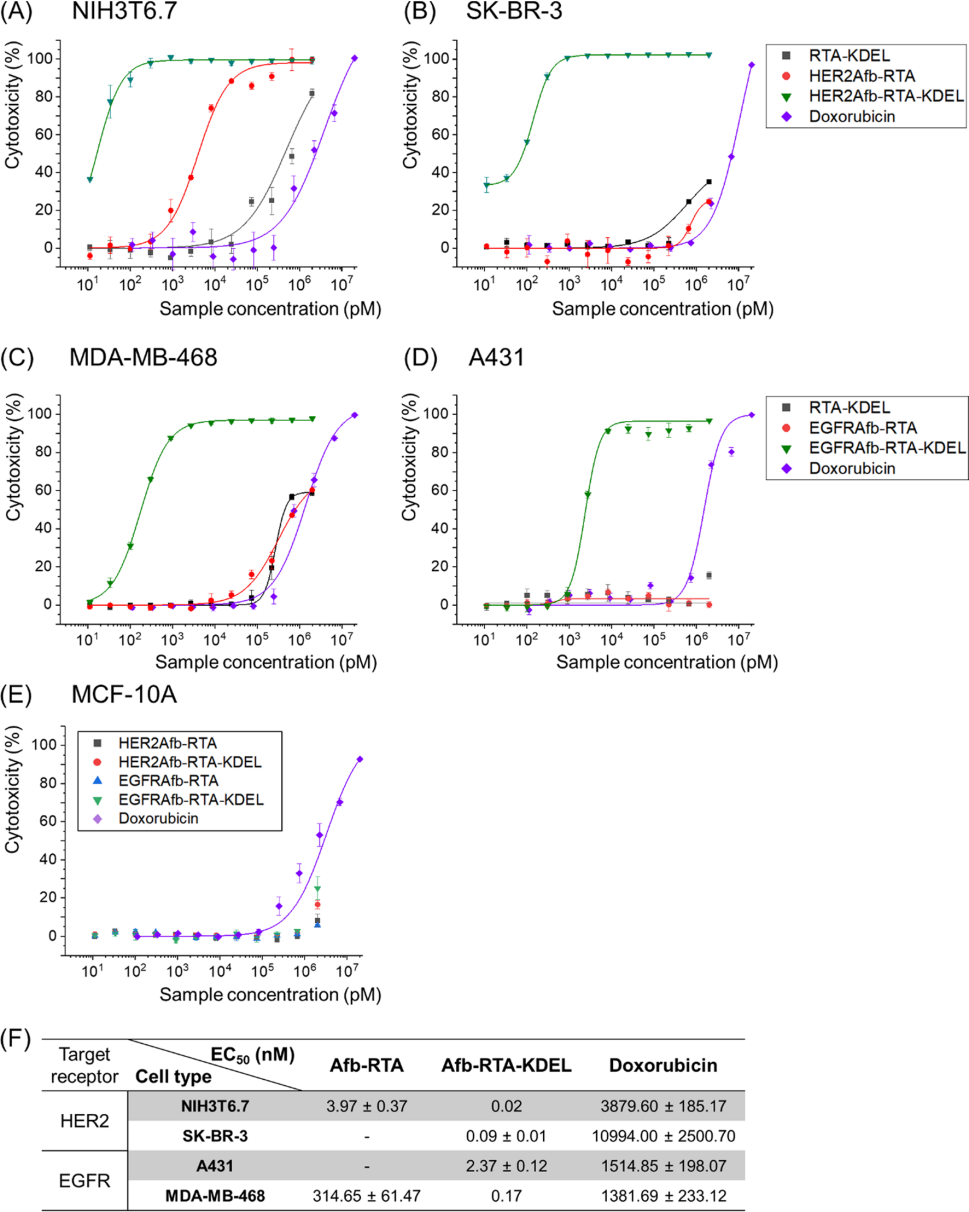
In vitro measurements of dose-dependent cytotoxicity. Dose-dependent cytotoxicity of RTA-KDEL, HER2Afb-RTA, HER2Afb-RTA-KDEL and doxorubicin in HER2-overexpressing **A** NIH3T6.7 and **B** SK-BR-3 cells; RTA-KDEL, EGFRAfb-RTA, EGFRAfb-RTA-KDEL and doxorubicin in EGFR-overexpressing **C** MDA-MB-468 and **D** A431 cells; HER2Afb-RTA, HER2Afb-RTA-KDEL, EGFRAfb-RTA, and EGFRAfb-RTA-KDEL in **E** MCF-10A, using CCK-8 assay. **F** Half-maximal effective concentration (EC_50_) values of HER2Afb-RTA, HER2Afb-RTA-KDEL, EGFRAfb-RTA, EGFRAfb-RTA-KDEL, and doxorubicin against each cell. Curves were fitted using a Hill equation to determine EC_50_ values. All data shown are means ± standard deviation (SD); n = 3

To restore the ER-targeting-capability, we genetically introduced the KDEL signal peptide [22, 23] at the C-termini of HER2Afb-RTA and EGFRAfb-RTA (Additional file 1: Fig. S2). We measured cell cytotoxicity in a dose-dependent manner with a cell counting kit-8 assay (Fig. 1A–E) and calculated EC_50_ values of each immunotoxin against its corresponding target cell [28] (Fig. 1F). As we hypothesized, addition of the KDEL signal peptide significantly improved the cytotoxicity of both the RITs (Fig. 1, green triangles vs. red circles). The EC_50_ value of HER2Afb-RTA-KDEL against NIH3T6.7 cells reduced from 3.97 to 0.02 nM, and that of EGFRAfb-RTA-KDEL against MDA-MB-468 cells reduced from 314.65 to 0.17 nM (Fig. 1). More surprisingly, both the KDEL-containing RITs showed dramatically enhanced cytotoxicity against SK-BR-3 and A431 cells which were resistant to the non-KDEL-containing RITs (Fig. 1, green triangles). However, MCF-10A, which is a non-tumorigenic normal epithelial cell line that neither overexpresses HER2 nor EGFR, did not exhibit any noticeable cell death upon treatment with the RITs, implying that their mode of action was cancer-specific (Fig. 1E). Cells were also treated with RTA-KDEL, without the targeting ligands, to investigate the effect of KDEL alone (Fig. 1A–D; black squares). RTA-KDEL showed moderate cytotoxicity against NIH3T6.7 cells, lower than that of HER2Afb-RTA and HER2Afb-RTA-KDEL. In the case of MDA-MB-468 cells, RTA-KDEL showed similar cytotoxicity to that of EGFRAfb-RTA (Fig. 1A and C; black squares and red circles). In contrast, RTA-KDEL did not show any noticeable cytotoxicity against SK-BR-3 and A431 cells, like HER2Afb-RTA and EGFRAfb-RTA (Fig. 1B and D; black squares and red circles). These results suggest that the cytotoxicity of RTA-KDEL is correlated with the target cell’s sensitivity toward HER2Afb-RTA or EGFRAfb-RTA. Although many cells have KDEL receptors on their surface, and RTA-KDEL may bind to them and direct the RTA to ER, SK-BR-3 and A431 cells were still resistant to RTA-KDEL implying that binding to KDEL receptors of SK-BR-3 and A431 cells would not be sufficient to kill them. Ricin-based RITs constructed here showed variable cytotoxicity toward different cell lines, but their cytotoxic efficacy was maximized when both the targeting ligand and the KDEL signal peptide were present.

### HER2Afb-RTA-KDEL and EGFRAfb-RTA-KDEL induce effective apoptosis of their corresponding target cancer cells

**Fig. 2:**
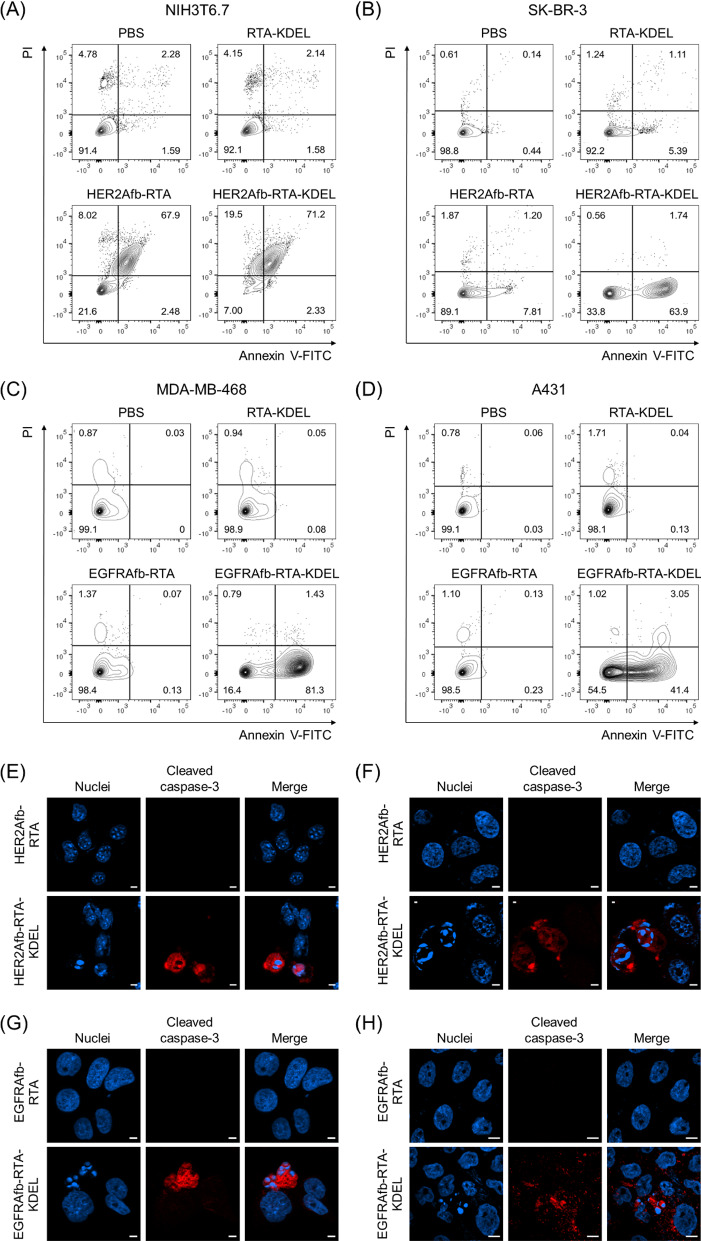
Flow cytometry and immunocytochemical analysis of cells treated with different RITs. **A** NIH3T6.7 and **B** SK-BR-3 cells were treated with PBS, RTA-KDEL, HER2Afb-RTA, or HER2Afb-KDEL. **C** MDA-MB-468 and **D** A431 cells were treated with PBS, RTA-KDEL, EGFRAfb-RTA, or EGFRAfb-RTA-KDEL. In each plot, the upper left and right quadrants represent necrosis and late apoptosis, respectively, and the lower left and right quadrants represent normal state of cells and early apoptosis, respectively. Relative populations of each quadrant are individually marked. Immunocytochemical detection of cleaved caspase-3 (red) and nuclei (blue) in: **E** NIH3T6.7 and **F** SK-BR-3 cells treated with HER2Afb-RTA or HER2Afb-RTA-KDEL; **G** MDA-MB-468 and **H** A431 cells treated with EGFRAfb-RTA or EGFRAfb-RTA-KDEL. Scale bars, 10 μm

Ricin holotoxin induces apoptosis by inactivating the ribosome and arresting protein synthesis in cells [10, 11]. We investigated whether the KDEL-containing RITs could induce target-specific apoptosis using Annexin V/propidium iodide (PI) staining, which measures apoptosis quantitatively based on the degree of cell membrane integrity. NIH3T6.7 and SK-BR-3 cells were challenged with 0.5 μM of RTA-KDEL, HER2Afb-RTA, or HER2Afb-RTA-KDEL; MDA-MB-468 and A431 cells were challenged with the same amounts of RTA-KDEL, EGFRAfb-RTA, or EGFRAfb-RTA-KDEL. Cells were then stained with annexin V-FITC and PI 48 h after sample treatment to prevent complete cell death in all populations. In all cell lines treated with phosphate-buffered saline (PBS), annexin V/PI double negative cells were dominant (Fig. 2A–D, lower left quadrants) indicating that the cells were alive. Either HER2Afb-RTA or HER2Afb-RTA-KDEL-treated NIH3T6.7 cells showed approximately 70% of annexin V/PI double positive populations, implying that the cells had undergone late apoptosis already (Fig. 2A, upper right quadrant). Consistent with the cell viability data, both HER2Afb-RTA and HER2Afb-RTA-KDEL effectively induced apoptosis of NIH3T6.7 cells regardless of the KDEL signal peptide. While 63.9% of SK-BR-3 cells treated with HER2Afb-RTA-KDEL were annexin V positive, but PI negative (lower right quadrants), indicating that they were in early apoptosis, only 7.81% shifted to an early apoptotic state upon HER2Afb-RTA treatment (Fig. 2B). Similarly, 81.3% of MDA-MB-468 cells treated with EGFRAfb-RTA-KDEL were in early apoptotic state, whereas 98.4% of cells were alive upon EGFRAfb-RTA or RTA-KDEL treatments (Fig. 2C). A431 cells also began to shift to early apoptotic phase only after the treatment of EGFRAfb-RTA-KDEL and 41.4% of cells were annexin V positive, but PI negative at 48 h incubation (Fig. 2D). MDA-MB-468 cells might shift to the apoptotic state more quickly than A431 cells, because MDA-MB-468 cells were more susceptible to EGFRAfb-RTA-KDEL than A431 cells (Fig. 1C, D, F; EC_50_ ~ 0.17 vs. 2.37 nM) and the flow cytometry analyses were performed at shorter timepoint (48 h) and lower sample concentration (0.5 μM) than the cell viability test to prevent complete cell death (Fig. 2).

To further confirm target-specific HER2Afb- or EGFRAfb-RTA-KDEL-induced apoptotic cell death at much lower concentration, cleaved caspase-3, a well-studied apoptosis marker, was visualized in the cells. Each cell line was treated with 5 nM of corresponding HER2Afb-RTA, EGFRAfb-RTA, HER2Afb-RTA-KDEL, or EGFRAfb-RTA-KDEL, and then stained with anti-cleaved caspase-3 antibody conjugated with a fluorescent dye (Fig. 2E–H). NIH3T6.7 and SK-BR-3 cells treated with HER2Afb-RTA-KDEL, and MDA-MB-468 and A431 cells treated with EGFRAfb-RTA-KDEL showed the fluorescent signals of cleaved caspase-3 (Fig. 2E–H, red) with fragmented nuclei, whereas there was no fluorescent signal or fragmented nuclei in other cells treated with either HER2Afb-RTA or EGFRAfb-RTA. These results suggest that RITs with the KDEL signal peptide (HER2Afb-RTA-KDEL and EGFRAfb-RTA-KDEL) successfully induced apoptosis in a target-dependent manner, leading to effective cancer cell death.

### KDEL signal peptide allows effective intracellular delivery of the RTA

**Fig. 3:**
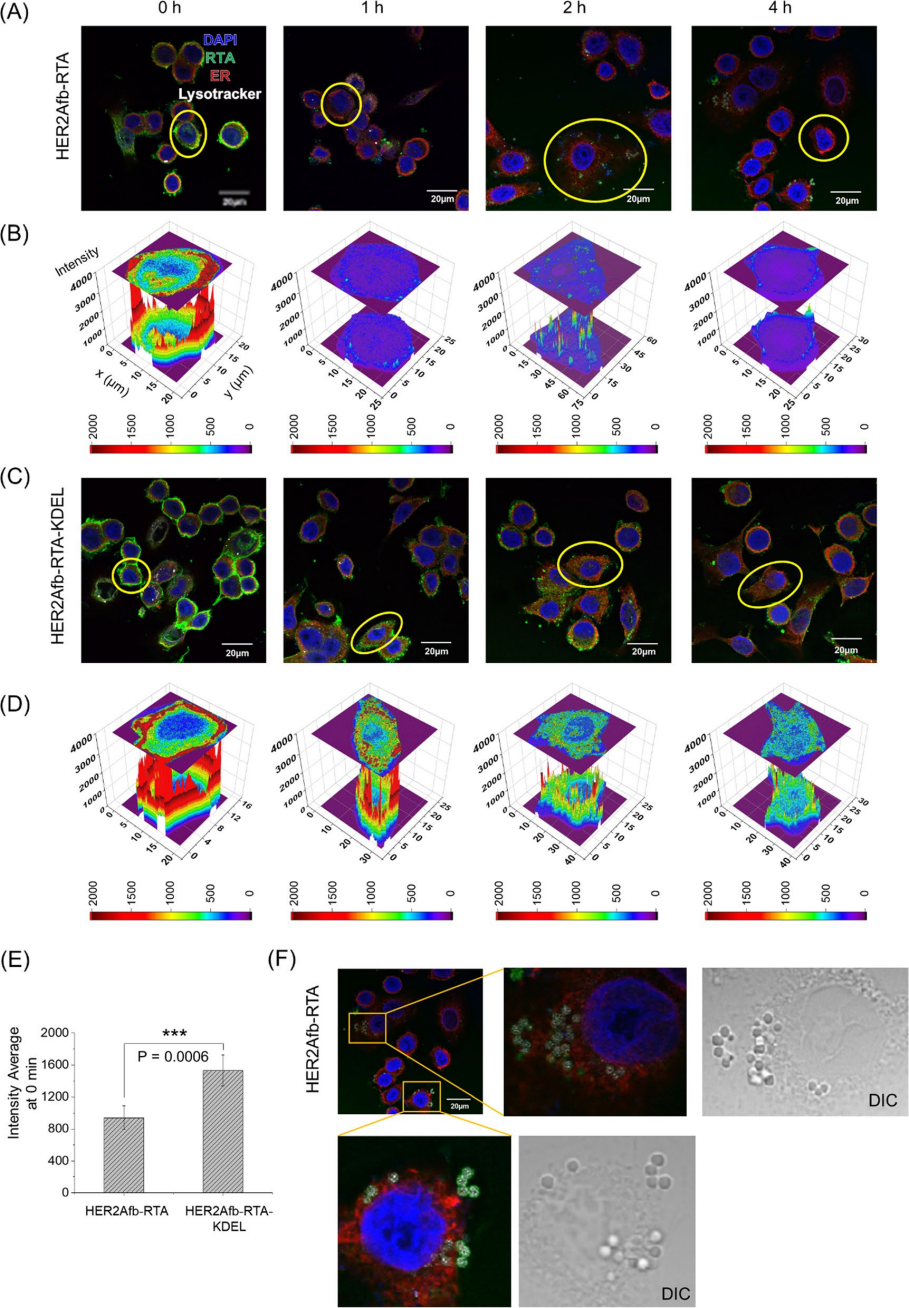
Fluorescence images of SK-BR-3 cells treated with **A** HER2Afb-RTA or **C** HER2Afb-RTA-KDEL at various time points. Nuclei, RTA, ER, and lysosomes are shown in blue, green, red, and white, respectively. Scale bars, 20 μm. One of the cells in **A** and **C** was selected and fluorescence intensity profiles of **B** HER2Afb-RTA and **D** HER2Afb-RTA-KDEL in the selected area were analyzed. The fluorescence intensity of RTA is represented by a colorimetric scale bar, and 2D projection of the area was drawn on top of the 3D plot. **E** Fluorescence signal intensity of HER2Afb-RTA or HER2Afb-RTA-KDEL bound to the cell surface at 0 h was statistically measured by line intensity analysis and plotted as a bar graph. All data are shown as means ± SD; ***p < 0.001. **F** Microvesicle-like structures containing RTA at the surface of SK-BR-3 cells treated with HER2Afb-RTA were enlarged with DIC images

To explore how the KDEL signal peptide enhances cytotoxicity, we first treated SK-BR-3 cells with either HER2Afb-RTA or HER2Afb-RTA-KDEL and visualized the intracellular localization of the RTA in each cell after various incubation times by fluorescence microscopy (Fig. 3, green in images). Both HER2Afb-RTA and HER2Afb-RTA-KDEL selectively bound to the surface of SK-BR-3 cells right after the treatment (Fig. 3A and C, 0 h). However, HER2Afb-RTA-KDEL bound more intensely than HER2Afb-RTA (Fig. 3E), probably due to surface-presented KDEL receptors on SK-BR-3 cells [29, 30]. HER2Afb-RTA dramatically disappeared from the cells with time, and was found outside the cells (Fig. 3A), whereas HER2Afb-RTA-KDEL was visualized inside the cells even after 4 h (Fig. 3C). Quantitative fluorescence analyses of the RTA revealed that most of the HER2Afb-RTA was removed from the cells within 1 h (Fig. 3B), whereas most of the HER2Afb-RTA-KDEL remained in the cytosol even after 4 h (Fig. 3D). Interestingly, we could observe budding microvesicle-like structures, which have the RTA and lysotracker signal together, only in SK-BR-3 cells treated with HER2Afb-RTA (Fig. 3A and F).

NIH3T6.7 cells were also treated with either HER2Afb-RTA or HER2Afb-RTA-KDEL and intracellular localization of the RTA was monitored. Similar to the observations in SK-BR-3 cells, HER2Afb-RTA-KDEL bound more strongly to NIH3T6.7 cell surface and remained longer in the cytosol than HER2Afb-RTA (Additional file 1: Fig. S3). However, unlike in SK-BR-3 cells, significant amounts of HER2Afb-RTA were still observed in the cytosol throughout the experiment and no microvesicle-like structures were observed in the cells (Additional file 1: Fig. S3A). These results imply that HER2Afb-RTAs selectively bind to HER2-overexpressing SK-BR-3, but they may be recycled back to the cell surface to be removed through microvesicle-like structures, making SK-BR-3 cells resistant to HER2Afb-RTA. However, NIH3T6.7 cells were not resistant to HER2Afb-RTA, probably because they lack a recycling machinery in the HER2-mediated endocytosis pathway, allowing HER2Afb-RTA to effectively enter the cells and induce apoptosis. The cytotoxicity in NIH3T6.7 cells may mainly depend on the initial amounts of the bound RITs rather than the presence of the KDEL signal peptide.

MDA-MB-468 cells were also treated with EGFRAfb-RTA or EGFRAfb-RTA-KDEL, to investigate targeting ligand-dependency of RITs with or without the KDEL signal peptide. EGFRAfb-RTA-KDEL also bound to the cell surface more and remained longer in the cytosol (Additional file 1: Fig. S4A–E), justifying our rationale that the addition of the KDEL signal peptide to immunotoxins would enhance their target cell-binding capacity and intracellular delivery efficiency, regardless of the targeting ligand species. Small amounts of EGFRAfb-RTA still remained inside MDA-MB-468 cells until 2 h post-treatment and disappeared completely by 4 h (Additional file 1: Fig. S4A and B), supporting the moderate cytotoxicity of EGFRAfb-RTA against these cells (Fig. 1C). We could also observe surface-budding microvesicles of RTA in MDA-MB-468 cells, like those in HER2Afb-RTA-treated SK-BR-3 cells (Additional file 1: Fig. S4F). These results imply that MDA-MB-468 cells also recycle EGFRAfb-RTA but less efficiently than SK-BR-3 cells, possibly resulting in their moderate sensitivity to EGFRAfb-RTA. These data strongly suggest that the KDEL signal peptide improves the cytotoxicity of both the RITs by circumventing a recycling pathway as well as directing them to the retrograde transport pathway to reach the ER to be delivered to the cytosol.

### KDEL signal peptide enables the RTA to circumvent endosomal recycling

**Fig. 4:**
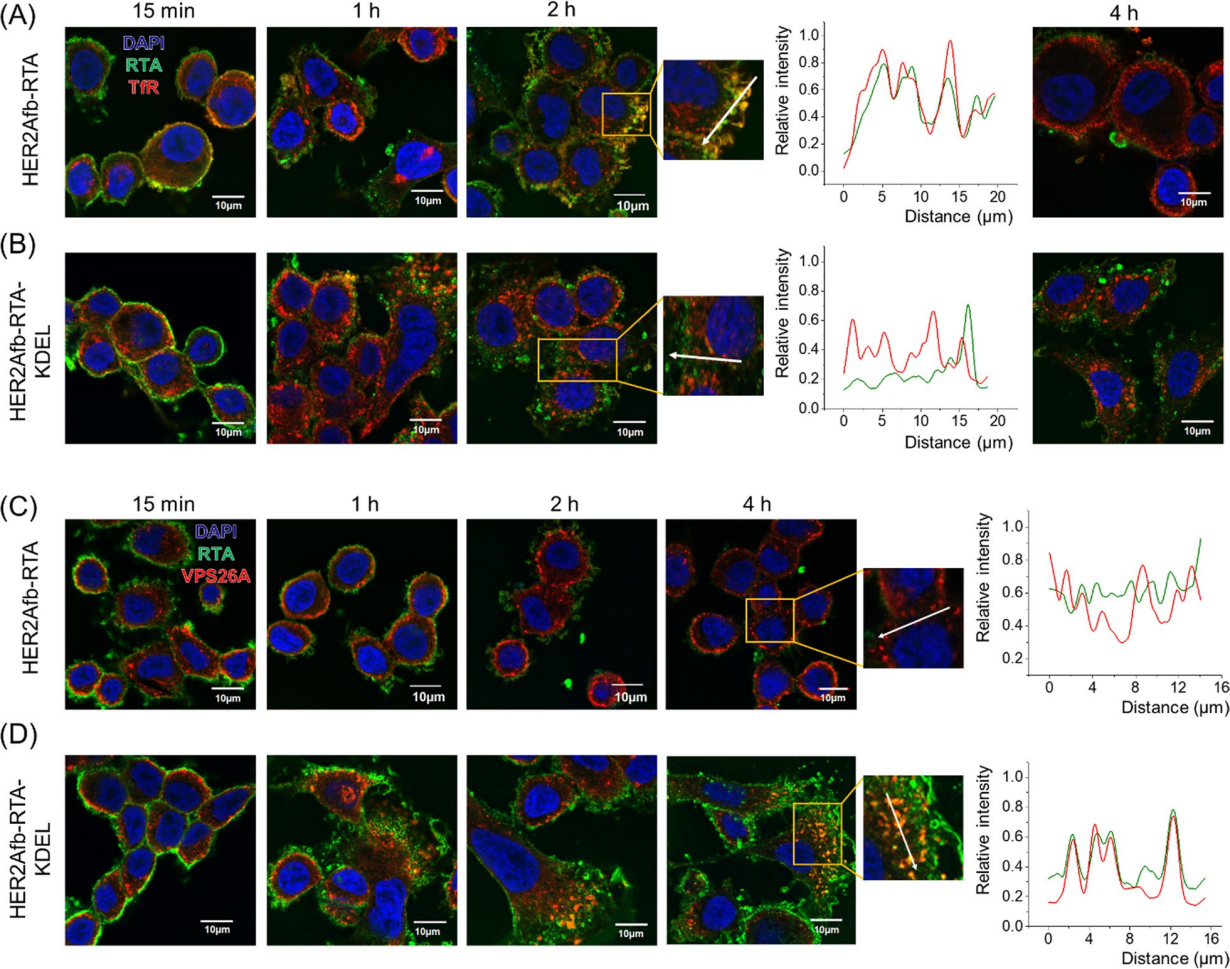
Fluorescence images of SK-BR-3 cells treated with HER2Afb-RTA (**A**, **C**) or HER2Afb-RTA-KDEL (**B**, **D**) at various time points. **A**, **B** Nuclei, RTA, and TfR are shown in blue, green, and red, respectively. **C**, **D** Nuclei, RTA, and VPS26A are shown in blue, green, and red, respectively. Co-localization of RTA and TfR (A, B at 2 h) or RTA and VPS26A (C, D at 4 h) was also analyzed with a line intensity profile according to distance. The analyzed lines are indicated as white arrows. Scale bars, 10 μm

To further investigate which pathway HER2Afb-RTA or HER2Afb-RTA-KDEL followed when HER2Afb-RTA-resistant cells, such as SK-BR-3 cells, were treated with them, we visualized the RTA in cells with a fluorescence microscope and compared their locations with transferrin receptors (TfR) [31] and VPS26A [32], which are recycling endosome and retromer markers, respectively. The fluorescent signals of HER2Afb-RTA (green) overlapped with those of TfR (red) 2 h after sample treatment, but completely disappeared 4 h after treatment (Fig. 4A); the fluorescent signals of HER2Afb-RTA-KDEL (green) and TfR (red) did not overlap, and both remained inside the cells even 4 h after treatment (Fig. 4B). These data suggest that the removal of HER2Afb-RTA observed in Fig. 3A is due to the recycling pathway acting between 2 and 4 h after sample treatment.

In contrast, we could observe the fluorescent signals of HER2Afb-RTA-KDEL (green) co-localizing with those of VPS26A (red) from 2 h post-treatment and lasting till 4 h after treatment (Fig. 4D). However, the fluorescent signals of HER2Afb-RTA (green) did not overlap with those of VPS26A, and they mostly disappeared after 4 h post-treatment (Fig. 4C). These results suggest that the addition of the KDEL signal peptide can overcome resistance of SK-BR-3 cells to HER2Afb-RTA, by switching from a recycling to a retrograde transport pathway, which seems to be a slower process (Fig. 4).

EGFRAfb-RTA and EGFRAfb-RTA-KDEL were also trafficked with TfR and VPS26A in MDA-MB-468 cells. Overlapping fluorescent signals of EGFRAfb-RTA (green) and TfR (red) were observed 2 h after sample treatment (Additional file 1: Fig. S5A). The fluorescent signals of EGFRAfb-RTA-KDEL (green) did not co-localize with those of TfR (red), but they overlapped with those of VPS26A (red) between 2 and 4 h after sample treatment. However, the fluorescent signals of EGFRAfb-RTA did not co-localize with those of VPS26A, again supporting the concept that the KDEL signal peptide re-routes the RTA to the retrograde pathway (Additional file 1: Fig. S5B).

### Albumin-binding domain and protein ligation system were introduced for in vivo anti-tumor experiment

Since HER2Afb-RTA-KDEL and EGFRAfb-RTA-KDEL showed excellent target-specific intracellular RTA delivery with low EC_50_ values in vitro, we decided to explore their in vivo efficacy as well. However, the small-sized RITs can be quickly cleared from circulation upon the systemic administration, before reaching the tumor site [33]. To increase blood circulation time and enhance in vivo therapeutic efficacy, we genetically introduced the previously developed albumin-binding domain (AlBD) [18, 19, 34–36] to the N-terminus of RTA-KDEL, constructing AlBD-RTA-KDEL. We and other groups previously showed that the AlBD-fused proteins bind with high affinity to human and mouse serum albumins in blood [15, 33], and stay approximately 10 times longer in blood than those without AlBD [33].

**Fig. 5:**
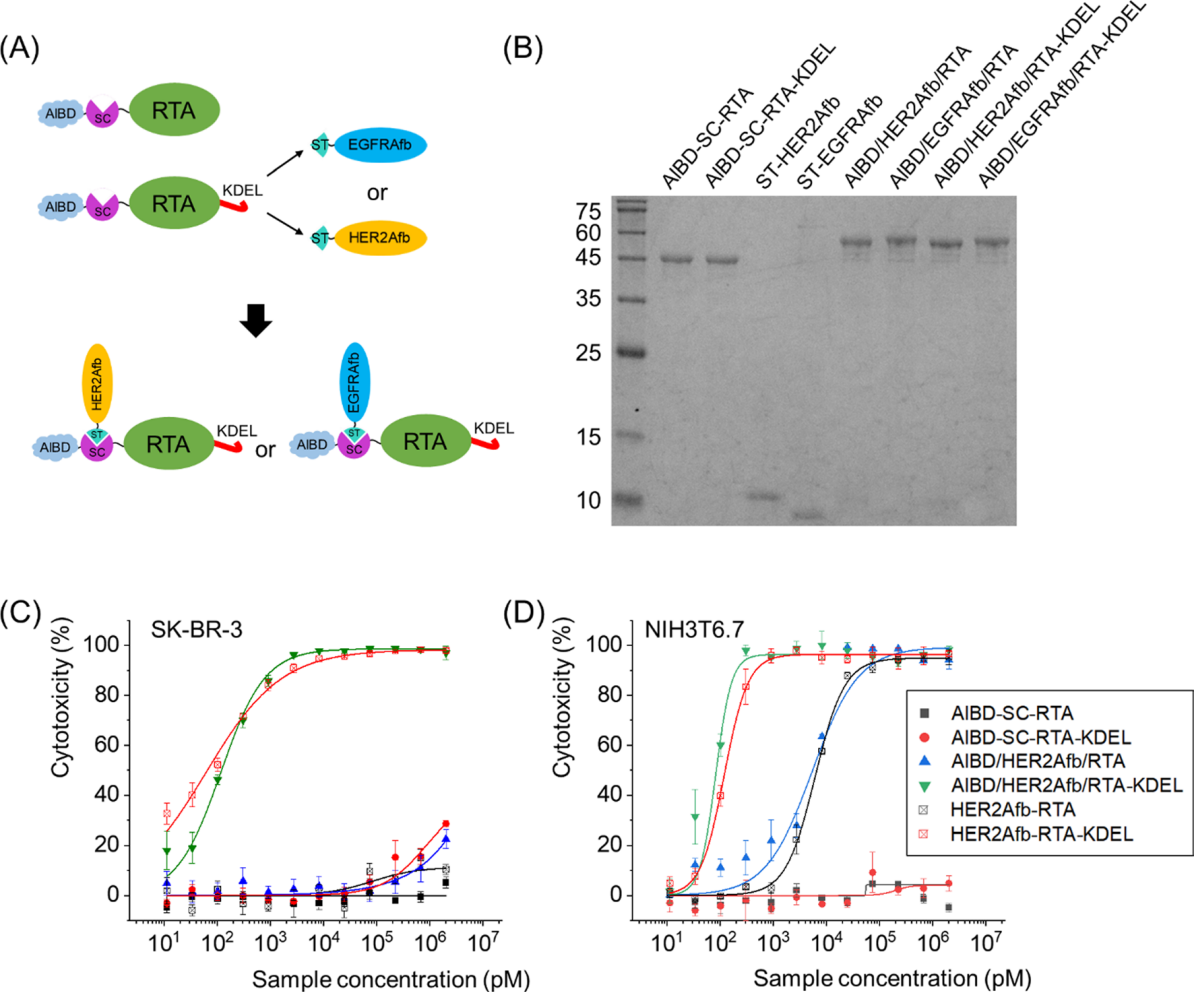
Construction of AlBD-SC-RTA-KDEL by genetic fusion of AlBD and SC, and subsequent target ligand addition via an SC/ST protein ligation system. **A** Schematic illustration and **B** SDS-PAGE analysis of post-translationally ligated resultants between AlBD-SC-RTA (50.0 kDa) and ST-HER2Afb (10.3 kDa) or ST-EGFRAfb (10.1 kDa), or between AlBD-SC-RTA-KDEL (50.4 kDa) and ST-HER2Afb or ST-EGFRAfb, forming AlBD/HER2Afb/RTA (60.3 kDa), AlBD/EGFRAfb/RTA (60.1 kDa), AlBD/HER2Afb/RTA-KDEL (60.7 kDa) and AlBD/EGFRAfb/RTA-KDEL (60.5 kDa), respectively. In vitro measurements of dose-dependent cytotoxicity of AlBD-containing RITs in **C** SK-BR-3 and **D** NIH3T6.7 cells, using CCK-8 assay. Each construct was indicated

In addition, we genetically inserted SpyCatcher protein between AlBD and the RTA as illustrated in Fig. 5A. The SpyCatcher (SC) and SpyTag (ST) are split forms of a fibronectin-binding protein derived from *Streptococcus* *pyogenes* and they form a spontaneous and irreversible isopeptide bond upon recognizing each other [37]. We can utilize this post-translational ligation between SC and ST to modularly assemble SC-fused RTA and ST-fused affibody molecules together. The usage of SC/ST would allow us to (i) bypass steric hindrance that AlBD exerts on the function of targeting ligand, (ii) give the toxin target-switchability, and (iii) adjust configuration of immunotoxins as Y-shape which makes them bulkier [18].

Newly constructed AlBD-SC-RTA-KDEL was successfully ligated with ST-fused HER2Afb or EGFRAfb to form AlBD/HER2Afb/RTA-KDEL or AlBD/EGFRAfb/RTA-KDEL (Fig. 5A and B). They exhibited almost identical target-specific cytotoxicity in vitro to the values measured for HER2Afb-RTA-KDEL or EGFRAfb-RTA-KDEL (Fig. 5C, D and Additional file 1: Fig. S6). AlBD-SC-RTA without the KDEL signal peptide was also constructed, ligated with either ST-fused HER2Afb or EGFRAfb, and examined as controls (Fig. 5B–D and Additional file 1: Fig. S6).

### AlBD/HER2Afb/RTA-KDEL significantly suppresses growth of tumor resistant to HER2Afb-RTA only

We established two in vivo mouse models, NIH3T6.7- and SK-BR-3-tumor-bearing mice, because they showed different responses to immunotoxins with the same targeting ligands with or without the KDEL signal peptide. We first investigated the anti-tumor effects of RTA variants on NIH3T6.7 tumor-bearing mice. NIH3T6.7 cells were allografted to immunodeficient nude mice and 5 μg of AlBD/HER2Afb/RTA-KDEL, AlBD/HER2Afb/RTA, AlBD-SC-RTA-KDEL or PBS were intravenously injected when the average tumor volume reached 100 mm^3^, approximately. Each group was injected every two or three days for eight times and body weights and tumor volumes were measured before injection (Additional file 1: Fig. S7A and B). Mice were sacrificed at day 28, and major organs (heart, lung, liver, kidney, and spleen) and tumor masses were biopsied (Additional file 1: Fig. S7C–E). Consistent with the in vitro data, AlBD/HER2Afb/RTA-KDEL and AlBD/HER2Afb/RTA noticeably suppressed the growth of NIH3T6.7 tumors, but AlBD/HER2Afb/RTA-KDEL exhibited a superior suppression effect without any significant side-effects to normal tissue (Additional file 1: Fig. S7). However, AlBD-SC-RTA-KDEL did not affect the growth of the tumors compared with controls (Additional file 1: Fig. S7A–D), suggesting that the targeting ligand is critical for in vivo efficacy. Although the in vivo tumor suppression efficacy of HER2Afb-RTA-KDEL and HER2Afb-RTA was statistically significant, the in vivo cytotoxic efficacy of both AlBD/HER2Afb/RTA-KDEL and AlBD/HER2Afb/RTA was not good enough to completely suppress tumor growth probably because in vitro efficacy is indeed not always translated to in vivo. The NIH3T6.7 tumors grew so quickly that AlBD/HER2Afb/RTA-KDEL was not potent enough to keep the tumors in check.

**Fig. 6:**
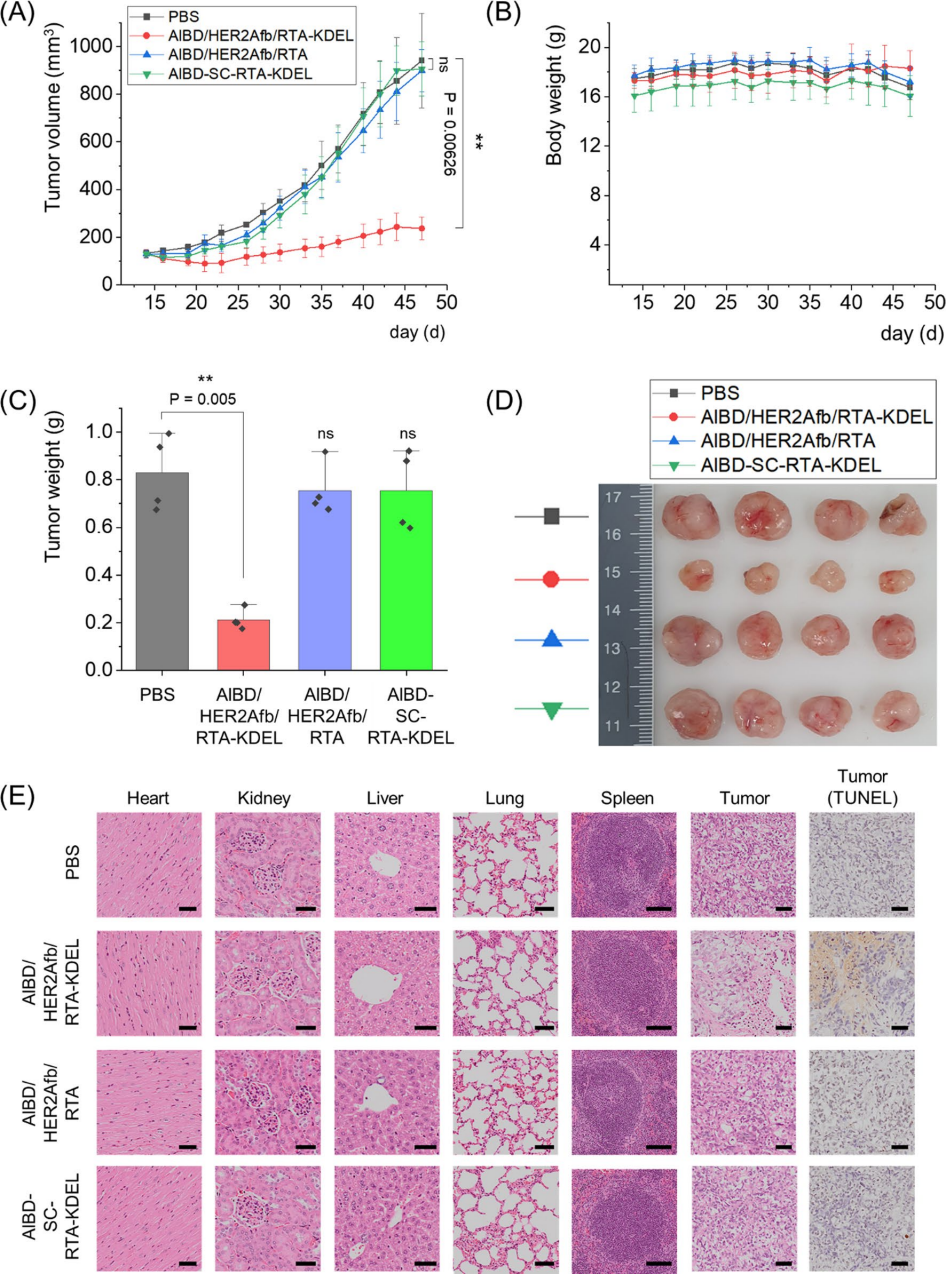
In vivo therapeutic efficacy of AlBD/HER2Afb/RTA-KDEL and other AlBD-containing RITs in SK-BR-3 tumor-bearing mice. **A** Tumor volumes and **B** body weights were measured with a caliper and scaler, respectively. **C** Weight of biopsied tumors were measured and **D** a picture of the biopsied tumors are shown. **E** The hearts, kidneys, livers, lungs, spleens, and tumors collected from sacrificed mice treated with PBS, AlBD/HER2Afb/RTA-KDEL, AlBD/HER2Afb/RTA, or AlBD-SC-RTA-KDEL were fixed, embedded in paraffin, and sectioned. The organ tissue and tumor sections were stained with hematoxylin and eosin (H&E) and extra tumor sections were independently stained with TUNEL. Images were captured using an Olympus virtual microscope. Scale bar, 100 μm at spleen and 50 μm at others. All data shown in **A**–**C** are the means ± SD; n = 4 per group; **p < 0.01; *ns* not significant

We next xenografted human breast cancer cells, SK-BR-3 cells, to immunodeficient nude mice and 5 μg of AlBD/HER2Afb/RTA-KDEL, AlBD/HER2Afb/RTA, AlBD-SC-RTA-KDEL or PBS were intravenously injected when the average tumor volume reached 130 mm^3^, approximately. Each group was injected every two or three days for eight times, and body weights and tumor volumes were measured before injection (Fig. 6A and B). Mice were sacrificed at day 47, and major organs (heart, lung, liver, kidney, and spleen) and tumor masses were biopsied (Fig. 6C–E). Similar to in vitro data, SK-BR-3 tumor growth was dramatically suppressed only upon the administration of AlBD/HER2Afb/RTA-KDEL, which has both the KDEL signal peptide and the targeting ligand; either AlBD/HER2Afb/RTA (without KDEL) or AlBD-SC-RTA-KDEL (without targeting ligand) did not influence SK-BR-3 tumor growth at all (Fig. 6A–D). There was no noticeable body weight change observed (Fig. 6B). Hematoxylin and eosin (H&E)-stained tissue sections of biopsied organs did not show any noticeable difference in morphology and structure of major organs among the different mice treatment groups, suggesting that there was no significant side-effect or off-target damage cause by RTA variants in major organs. However, H&E-stained tumor sections of the mice treated with AlBD/HER2Afb/RTA-KDEL showed a much loosely packed structure compared with the other tumor sections (Fig. 6E). Furthermore, terminal deoxynucleotidyl transferase dUTP nick end labeling (TUNEL) assay of tumor sections of the mice treated with AlBD/HER2Afb/RTA-KDEL also showed high levels of fragmented DNA (Fig. 6E, brown stain), whereas no noticeable DNA fragmentation was observed in the tumor sections of mice from other treatment groups (Fig. 6E). Taken together, these results implicate that AlBD/HER2Afb/RTA-KDEL circulated in the blood stream of the mice for a long time, specifically delivered to the SK-BR-3 tumor site, successfully entered the cells, and effectively suppressed SK-BR-3 tumor growth by inducing apoptotic cell death. Effective tumor suppression was possible only when the targeting ligand and the KDEL signal peptide simultaneously existed with the RTA.

**Fig. 7:**
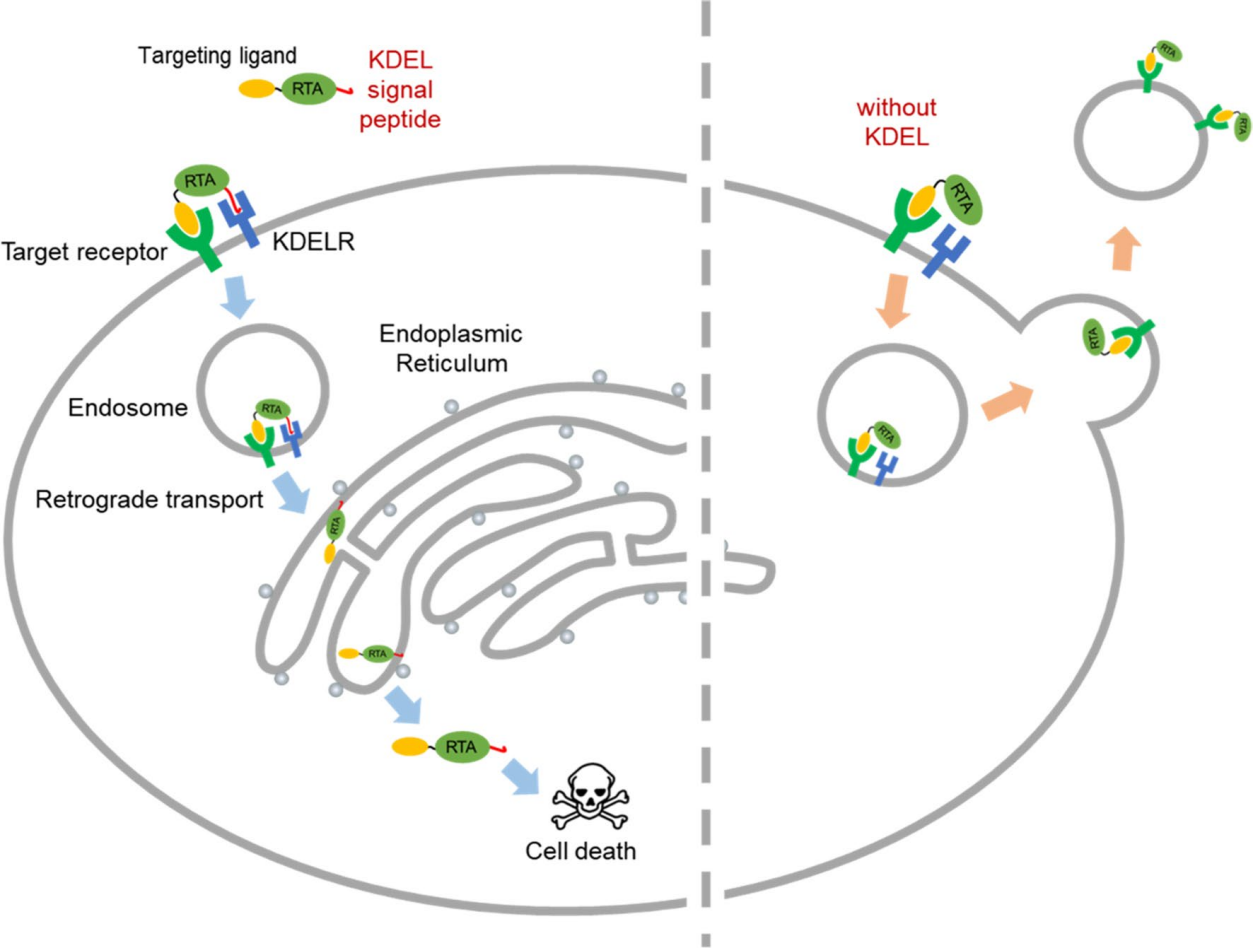
A proposed model for intracellular RTA delivery with or without KDEL signal peptide in RTA-resistant cells

There have been numerous reports on ricin-based RITs [38–40]. Although they have been actively developed against Hodgkin and non-Hodgkin lymphoma including clinical studies [41–44] as well as solid tumors preclinically [45–47], their anti-cancer efficacy was rather moderate. When the RTB was replaced with another targeting moiety, ricin-based RITs were less efficient because the RTB has roles in intracellular routing to the ER as well as binding to cell surface galactoses [3, 40]. On the other hand, the RITs with the holotoxin were often less specific than those with the RTA alone, resulting in off-target effects [3]. Based on our results, we propose a working model for intracellular delivery of ricin-based RITs constructed in this study. When both the targeting ligand and the KDEL signal peptide are fused to the RTA, it undergoes receptor-mediated endocytosis upon cancer-cell binding, follows a retrograde transport pathway guided by the KDEL signal peptide to reach the ER [8], and is subsequently released to the cytosol by the ERAD pathway [3, 9], resulting in apoptotic cell death (Fig. 7, left half). In contrast, the RTA with the targeting ligand alone can still be endocytosed, but most of them will be recycled back to the cell surface to be removed from the cells, resulting in decreased therapeutic efficacy (Fig. 7, right half).

Although it has been known that the KDEL signal peptide directs protein toxins to the ER and improves the cytotoxicity of the RTA [23, 48, 49], most previous studies demonstrated their cytotoxic efficacy in vitro rather than in vivo. Here, we constructed ricin-based RITs using both affibody molecule-based targeting ligands and the KDEL signal peptide. We successfully showed their excellent target-specific cytotoxic efficacy in vivo with the aid of albumin-binding domain. These findings offer a potential strategy to increase therapeutic efficacy of protein-based immunotoxins by rationally designing intracellular routes and incorporating guiding sequences to overcome the drug tolerance of tumors.

## Conclusions

Here, we genetically combined the RTA, affibody molecules, and the KDEL signal peptide to construct two different types of ricin-based RITs: HER2Afb-RTA-KDEL and EGFRAfb-RTA-KDEL. They selectively bound to their corresponding target cancer cells, NIH3T6.7 and SK-BR-3 cells or MDA-MB-468 and A431 cells, respectively, and effectively killed them with extremely low EC_50_ values. Flow cytofluorimetric analyses using Annexin V/PI staining and the cleaved caspase-3 immunocytochemical analyses of RIT-treated target cancer cells revealed that they successfully induced target-specific cellular apoptosis, leading to effective cancer cell death.

Upon the selective binding of the RITs to their corresponding target cancer cells, they were endocytosed and the RTAs were subsequently delivered to the cytosol through the retrograde transport pathway, avoiding the alternate recycling pathway of HER2Afb-RTA- or EGFRAfb-RTA-resistant cancer cells. The translational implications of these RITs were further evaluated by using SK-BR-3 tumor-bearing mice and additional construct modifications. To improve in vivo therapeutic efficacy and adopt target-switchability, AlBD and SC proteins were further introduced. AlBD/HER2Afb/RTA-KDEL was finally constructed and systematically administered to the tumor-bearing mice, allowing specific delivery to the SK-BR-3 tumor sites and subsequent intracellular delivery of the RTA to cancer cells, resulting in effective tumor suppression by the RTA, inducing apoptotic cell death without any noticeable side-effects.

The strategy of developing multi-component RITs described here may offer new possibilities to develop “magic bullets” that can effectively suppress the growth of tumors which are resistant to conventional immunotoxins and anti-cancer drugs.

## Methods

### Cloning and purification of proteins

Gene of RTA was a gift from Vern Schramm (Addgene plasmid #64,029; http://n2t.net/addgene:64029; RRID: Addgene_64029) [50]. To make HER2Afb-RTA and EGFRAfb-RTA, RTA gene was inserted into a pETDuet-1 vector containing HER2Afb or EGFRAfb gene with the C-terminal his-tag. KDEL signal peptide (KDEL) was genetically added to the C-termini of HER2Afb-RTA or EGFRAfb-RTA. The genes encoding albumin-binding domain (AlBD) [18] and SpyCatcher (SC) were assembled with RTA and RTA-KDEL in pETDuet-1 vectors by the Gibson assembly to construct AlBD-SC-RTA and AlBD-SC-RTA-KDEL, respectively.

The plasmids encoding each recombinant protein were individually transformed into Escherichia coli, BL21 (DE3) strain and the recombinant proteins were overexpressed by inducing with isopropyl β-D-1-thiogalactopyranoside (IPTG) at 18 ℃ overnight. The cultured bacterial cells were harvested, lysed by lysozyme, sonicated, and supernatant containing soluble protein extracts were obtained by centrifugation. The protein extracts were loaded onto batch columns (Bio-Rad) filled with Ni Sepharose 6 fast flow (Cytiva) for immobilized metal affinity chromatography (IMAC). His-tagged proteins were captured by the resin and eluted by an elution buffer (20 mM Na2HPO4, 500 mM NaCl, 1 M imidazole, pH 7.4). The eluted fractions that contain proteins we want were analyzed with SDS-PAGE and the purity and apparent molecular weights of them were determined. Fractionated proteins were collected and dialyzed against PBS extensively to remove imidazole and other components. Purified protein concentrations were measured with Pierce™ BCA Protein Assay Kit (Thermo Fisher).

### Cell culture

NIH3T6.7 cell line, an engineered mouse fibroblast cell, was generously gifted from Prof. Jeongsoo Yoo, Kyungpook National University, South Korea. A431 (human skin cancer cell), SK-BR-3 (human breast cancer cell), MDA-MB-468 (human breast cancer cell), and MCF-10A cells (non-tumorigenic human epithelial cell) were purchased from ATCC. NIH3T6.7, A431, and MDA-MB-468 cells were cultured with DMEM (Gibco), SK-BR-3 cells were cultured with RPMI1640 (Gibco), and MCF-10A was cultured with DMEM/F-12 (Gibco). DMEM and RPMI640 were supplemented with 10% FBS (Gibco) and 1% antibiotic–antimycotic (Gibco). DMEM/F-12 was supplemented with 5% horse serum (Gibco), 1% antibiotic–antimycotic, 20 ng/ml of human EGF (Prospec Bio), 0.5 mg/ml of hydrocortisone (Abcam), and 10 μg/ml of human insulin (Abcam). All cells were incubated in a humidified incubator (Thermo Fisher) at 37 ℃ and 5% of CO_2_.

### Cell viability test

To assess target-specific cytotoxicity of HER2Afb-RTA, EGFRAfb-RTA, HER2Afb-RTA-KDEL or EGFRAfb-RTA-KDEL quantitatively, 5000 cells of each cell line were seeded each well of 96 well plates (Eppendorf) overnight. Subsequently, cells were challenged with 20 μM of doxorubicin hydrochloride (TCI) or 2 μM of RTA-KDEL, HER2Afb-RTA, EGFRAfb-RTA, HER2Afb-RTA-KDEL, or EGFRAfb-RTA-KDEL with a series of threefold dilutions for 1 h, washed with PBS to remove unbound samples, fed with corresponding culture media, and incubated for 3 days further. Viability of the cells was measured with a cell counting kit-8 (CCK-8) assay (Dojindo). CCK-8 reagent mix solution was added onto each well, incubated for 2–4 h, and the absorption of each well was measured at 450 nm using a spectrophotometer.

### Annexin V/PI staining and flow cytometry analysis

500,000 cells of each cell line were seeded each well of 6 well plates (Thermo Fisher) and incubated overnight in a humidified incubator at 37 °C and 5% of CO_2_. Subsequently, NIH3T6.7 and SK-BR-3 cells were challenged with PBS, 0.5 μM of RTA-KDEL, HER2Afb-RTA, or HER2Afb-RTA-KDEL. MDA-MB-468 and A431 were also challenged with PBS, 0.5 μM of RTA-KDEL, EGFRAfb-RTA, or EGFRAfb-RTA-KDEL. After 1 h incubation, cells were washed with clean PBS twice, filled with 2 mL of complete media, and incubated for additional 48 h. Subsequently, cells were treated with trypsin–EDTA solution (Gibco), harvested, washed, and stained with dead cell apoptosis kit (Invitrogen). Cells were resuspended with annexin V-FITC and propidium iodide solution, and then analyzed by flow cytometry (BD LSR fortessa).

### Immunocytochemistry

20,000 cells of NIH3T6.7 and 40,000 cells of SK-BR-3, MDA-MB-468, and A431 were seeded on the coverslip in 24 well plate (SPL) and incubated overnight in the humidified cell incubator. NIH3T6.7 and SK-BR-3 cells were treated with 5 nM of HER2Afb-RTA or HER2AFb-RTA-KDEL. MDA-MB-468 and A431 were treated with 5 nM of EGFRAfb-RTA or EGFRAfb-RTA-KDEL. After 1 h treatments, cells were replaced with fresh media and incubated at least 8 h. Cells were washed with PBS, fixed with 4% paraformaldehyde (Electron Microscopy Sciences) in PBS for 10 min at room temperature and permeabilized in 0.2% Triton X-100 containing PBS (PBST) for 10 min. Fixed cells were blocked in 5% normal goat serum (Thermo Fisher) for 30 min at room temperature and incubated overnight at 4 ℃ with anti-cleaved caspase-3 antibody (Cell signaling technology, #9964) diluted in 5% goat serum in PBST. The cells were washed three times with PBST and incubated for 1 h at room temperature with anti-rabbit antibody conjugated with Alexa 594 dye (Thermo Fisher, R37117). Nuclei were stained with 1 μg/ml Hoechst for 10 min, and coverslips were mounted with ProLong Diamond Antifade Mountant (Thermo Fisher). Images were taken using a fluorescence microscope (Carl Zeiss AxioObserver 7).

### Confocal microscopic imaging

150,000 cells were counted and seeded on the coverslip in 12 well plates (Thermo Fisher) overnight. NIH3T6.7 and SK-BR-3 cells were treated with 1 µM of either HER2Afb-RTA or HER2Afb-RTA-KDEL and MDA-MB-468 cells were treated with 1 µM of either EGFRAfb-RTA or EGFRAfb-RTA-KDEL and they were cultured for 0 (or 15 min), 1, 2, and 4 h. Cells were washed and filled with new media after indicated culture times. Lysotracker RND-99 (Thermo Fisher) was added to each well 30 min before fixation. All cells were fixed and permeabilized with BD cytofix/cytoperm kit and blocked with cytoperm buffer containing 3% BSA. To visualize RTA, cells were treated with anti-RTA antibody (Kerafast, EMT011) for 1 h, washed, and stained with Alexa Fluor Plus 488 dye-conjugated anti-mouse goat secondary antibody (Thermo Fisher, A32723) for 1 h. After multiple washings, ProLong Diamond Antifade Mountant with DAPI (Thermo Fisher) was used for nuclear visualization.

To visualize subcellular components, anti-calnexin antibody for endoplasmic reticulum (Abcam, ab22595), anti-transferrin receptor antibody for recycling endosome (Abcam, ab84036), and anti-VPS26A antibody for retromer (Abcam, ab23892) were used. These antibodies were stained with Alexa flour Plus 647 dye-conjugated anti-rabbit goat secondary antibody (Thermo Fisher, A32733). All images were obtained with a FV-1000 confocal microscope (Olympus). The fluorescent signals of RTA variants bound at cell surface were selected by drawing lines along cell membrane of all cells, and the fluorescent intensity of the designated line was analyzed by ROI (region of interest) measurement of a FV10-ASW software (Olympus). The fluorescent intensity profiles of designated cell area were also analyzed by the same software. To investigate co-localization between RTA variants and TfR or VPS26A, ROI lines were drawn across one of cells and the fluorescent intensity of each component was measured.

### In vivo anti-tumor experiments & H&E staining

Nude mice at 4–5 weeks of age were purchased from Orient Bio. For a SK-BR-3 xenograft model, 20 million of SK-BR-3 cells were xenografted at the right flank of the mice with matrigel (Corning). After the volume of SK-BR-3 tumor reaches approximately 130 mm^3^, 6 µg of AlBD-SC-RTA-KDEL, AlBD/HER2Afb/RTA, or AlBD/HER2Afb/RTA-KDEL were administrated intravenously for each group (100 µl each) for 8 times in 2 or 3-day intervals. Tumor volumes and body weights were measured with a caliper and weighing scale, respectively, and actual final weights of tumor masses were assessed by biopsy at day 47. Major organs (liver, kidney, spleen, heart, and lung) were also biopsied simultaneously and fixed with 10% formalin (Sigma-Aldrich). The biopsied and fixed organs and tumor masses were made into paraffin blocks, sectioned, and stained with hematoxylin and eosin (H&E). DNA fragmentation in the paraffin section of the tumor masses were stained by TUNEL assay, to monitor apoptosis occurred in the tumors. H&E staining and TUNEL assays was done with the aid of LABCORE, Inc.

### Statistical analysis

Microsoft Excel was utilized to calculate average ± standard deviation (Figs. 1, 3E, 5C, D, 6A–C, Additional file 1: Figs. S1, S3E, S4E, S6, and S7A–C) and two-sample Student’s t-test for P-values (Figs. 3E, 6A, C, Additional file 1: Figs. S3E, S4E, S7A, C). Standard curves of cell viability assay were fitted by Hill equation using Origin 2020 (OriginLab) to determine EC_50_ values of each experimental group. (Figs. 1, 5C, D, Additional file 1: Figs. S1 and S6). In vivo tumor volume and body weight data was drawn as a line graph (Fig. 6A, B and Additional file 1: Fig. S7A, B) with Origin 2020, too.

## Supplementary Information

**Additional file 1: Figure S1.** In vitro quantitative measurements of dose-dependent cytotoxicity of HER2Afb-RTA or doxorubicin to (A) NIH3T6.7 and (B) SK-BR-3 cells and EGFRAfb-RTA, or doxorubicin to (C) MDA-MB-468 and (D) A431 cells. HER2Afb-RTA and EGFRAfb-RTA exhibited moderate or low cytotoxicity against target cells, compared to doxorubicin. All data shown are means ± standard deviation; n = 3. Curves are fitted using a Hill equation. **Figure S2.** Amino acid sequence information of (A) HER2Afb-RTA and HER2Afb-RTA-KDEL and (B) EGFRAfb-RTA and EGFRAfb-RTA-KDEL. The sequences of HER2Afb and EGFRAfb are indicated in blue and that of RTA is indicated in red. Linkers, his-tag, and KDEL signal peptide are indicated in black. (C) HER2Afb-RTA, HER2Afb-RTA-KDEL, EGFRAfb-RTA, EGFRAfb-RTA-KDEL, and RTA-KDEL were purified with IMAC and analyzed by SDS-PAGE. **Figure S3.** Fluorescence microscopic images of NIH3T6.7 cells treated with (A) HER2Afb-RTA or (C) HER2Afb-RTA-KDEL at various times. Nuclei, RTA, ER, and lysosomes are shown in blue, green, red, and white, respectively. Scale bars, 20 μm. One of cells in (A) and (C) was selected and fluorescent intensity profiles of (B) HER2Afb-RTA and (D) HER2Afb-RTA-KDEL in the selected area were analyzed. The fluorescent intensity of RTA is represented by colorimetric scale bar, and 2D projection of the area was drawn at the top of the 3D plot. (E) Fluorescent signal intensity of HER2Afb-RTA or HER2Afb-RTA-KDEL bound to the cell surface at 0 h was statistically measured by line intensity analysis and plotted as a bar graph. Data shown in the bar graph are the means ± SD; ****p < 0.0001. **Figure S4.** Fluorescence microscopic images of MDA-MB-468 cells treated with (A) EGFRAfb-RTA or (C) EGFRAfb-RTA-KDEL at various times. Nuclei, RTA, ER, and lysosomes are shown in blue, green, red, and white, respectively. Scale bars, 20 μm. One of cells in (A) and (C) was selected and fluorescent intensity profiles of (B) EGFRAfb-RTA and (D) EGFRAfb-RTA-KDEL in the selected area were analyzed. The fluorescent intensity of RTA is represented by colorimetric scale bar, and 2D projection of the area was drawn at the top of the 3D plot. (E) Fluorescent signal intensity of EGFRAfb-RTA or EGFRAfb-RTA-KDEL bound to the cell surface at 0 h was statistically measured by line intensity analysis and plotted as a bar graph. (F) Microvesicle-like structures containing RTA at the surface of MDA-MB-468 treated with EGFRAfb-RTA were enlarged with DIC images. Data shown in the bar graph are the means ± SD; *p < 0.05. **Figure S5.** Fluorescence microscopic images of MDA-MB-468 cells treated with EGFRAfb-RTA (A, C) or EGFRAfb-RTA-KDEL (B, D) at various times. (A, B) Nuclei, RTA, and TfR were shown in blue, green, and red, respectively. (C, D) Nuclei, RTA, and VPS26A were shown in blue, green, and red, respectively. Co-localization of RTA and TfR (A, B at 2 h) or RTA and VPS26A (C, D at 4 h) was also analyzed with a line intensity profile according to distance. The analyzed lines are indicated as white arrows. Scale bars, 10 μm. **Figure S6.** In vitro quantitative measurements of dose-dependent cytotoxicity of RTA variants to (A) MDA-MB-468 and (D) A431 cells with CCK-8 assay. All data shown are means ± standard deviation; n = 3. Curves are fitted using a Hill equation. **Figure S7.** In vivo therapeutic efficacy of AlBD/HER2Afb/RTA-KDEL and other RTA variants against NIH3T6.7 tumor-bearing mice. NIH3T6.7 cells were allografted to nude mice on day 0. After the tumor volume reached approximately 100 mm^3^, PBS, AlBD/HER2Afb/RTA-KDEL, AlBD/HER2Afb/RTA, and AlBD-SC-RTA-KDEL (5 μg/100 μl each) were intravenously injected for a total of eight times in 2 or 3 day intervals. (A) tumor volumes and (B) body weights were measured with a caliper and scaler, respectively. (C) Weight of biopsied tumors were measured and (D) picture of the biopsied tumors are shown. (E) The hearts, kidneys, livers, lungs, spleens, and tumors collected from sacrificed mice treated with PBS, AlBD/HER2Afb/RTA-KDEL, AlBD/HER2Afb/RTA, and AlBD-SC-RTA-KDEL were fixed, embedded in paraffin, and sectioned. The organ tissue and tumor sections were stained with H&E. Images were captured using an Olympus virtual microscope. Scale bar, 50 μm. All data shown in (A-C) are the means ± SD; n = 5 per group; *p < 0.05; **p < 0.01; ***p < 0.001.

## Data Availability

The datasets used during the current study are available from the corresponding author on reasonable request.

## Abbreviations

RTA: Ricin A chain
RTB: Ricin B chain
RIT: Recombinant immunotoxin
KDEL: Lys-Asp-Glu-Leu
HER2: Human epidermal growth factor receptor 2
EGFR: Epidermal growth factor receptor
SC: SpyCatcher
ST: SpyTag
Afb: Affibody
AlBD: Albumin binding domain
PBS: Phosphate-buffered saline
TfR: Transferrin receptor
VPS26A: Vacuolar protein sorting-associated protein 26A
ER: Endoplasmic reticulum
ERAD: ER-associated protein degradation
PI: Propidium iodide
SDS-PAGE: Sodium dodecyl sulfate–polyacrylamide gel electrophoresis
CCK-8: Cell counting kit-8

## References

[1] Smaglo BG, Aldeghaither D, Weiner LM (2014). The development of immunoconjugates for targeted cancer therapy. Nat Rev Clin Oncol.

[2] Pastan I, Hassan R, FitzGerald DJ, Kreitman RJ (2006). Immunotoxin therapy of cancer. Nat Rev Cancer.

[3] Słomińska-Wojewódzka M, Sandvig K (2013). Ricin and ricin-containing immunotoxins: insights into intracellular transport and mechanism of action in vitro. Antibodies.

[4] Manoukian G, Hagemeister F (2009). Denileukin diftitox: a novel immunotoxin. Expert Opin Biol Ther.

[5] Balint G (1974). Ricin: the toxic protein of castor oil seeds. Toxicology.

[6] Odumosu O, Nicholas D, Yano H, Langridge W (2010). AB toxins: a paradigm switch from deadly to desirable. Toxins.

[7] Rutenber E, Ready M, Robertus JD (1987). Structure and evolution of ricin B chain. Nature.

[8] Day PJ, Owens SR, Wesche J, Olsnes S, Roberts LM, Lord JM (2001). An interaction between ricin and calreticulin that may have implications for toxin trafficking. J Biol Chem.

[9] Lord JM, Spooner RA (2011). Ricin trafficking in plant and mammalian cells. Toxins.

[10] Endo Y, Tsurugi K (1987). RNA N-glycosidase activity of ricin A-chain. Mechanism of action of the toxic lectin ricin on eukaryotic ribosomes. J Biol Chem.

[11] Tesh VL (2011). The induction of apoptosis by Shiga toxins and ricin. Curr Top Microbiol Immunol.

[12] Weigel KJ, Shen L, Thomas CL, Alber D, Drapalik L, Schafer ZT, Lee SW (2015). Design and evaluation of a peptide-based immunotoxin for breast cancer therapeutics. FEBS Open Bio.

[13] Sánchez-García L, Serna N, Álamo P, Sala R, Céspedes MV, Roldan M, Sánchez-Chardi A (2018). Self-assembling toxin-based nanoparticles as self-delivered antitumoral drugs. J Control Release.

[14] Akbari B, Farajnia S, Ahdi Khosroshahi S, Safari F, Yousefi M, Dariushnejad H, Rahbarnia L (2017). Immunotoxins in cancer therapy: review and update. Int Rev Immunol.

[15] Dhillon S (2018). Moxetumomab pasudotox: first global approval. Drugs.

[16] Deng C, Xiong J, Gu X, Chen X, Wu S, Wang Z, Wang D, Tu J, Xie J (2017). Novel recombinant immunotoxin of EGFR specific nanobody fused with cucurmosin, construction and antitumor efficiency in vitro. Oncotarget.

[17] Sokolova E, Proshkina G, Kutova O, Shilova O, Ryabova A, Schulga A, Stremovskiy O (2016). Recombinant targeted toxin based on HER2-specific DARPin possesses a strong selective cytotoxic effect in vitro and a potent antitumor activity in vivo. J Control Release.

[18] Park SG, Choi B, Bae Y, Lee YG, Park SA, Chae YC, Kang S (2020). Selective and effective cancer treatments using target-switchable intracellular bacterial toxin delivery systems. Adv Therap.

[19] Altai M, Liu H, Orlova A, Tolmachev V, Gräslund T (2016). Influence of molecular design on biodistribution and targeting properties of an Affibody-fused HER2-recognising anticancer toxin. Int J Oncol.

[20] Yu X, Yang Y-P, Dikici E, Deo SK, Daunert S (2017). Beyond antibodies as binding partners: the role of antibody mimetics in bioanalysis. Annu Rev Anal Chem.

[21] Löfblom J, Feldwisch J, Tolmachev V, Carlsson J, Ståhl S, Frejd FY (2010). Affibody molecules: engineered proteins for therapeutic, diagnostic and biotechnological applications. FEBS Lett.

[22] Denecke J, De Rycke R, Botterman J (1992). Plant and mammalian sorting signals for protein retention in the endoplasmic reticulum contain a conserved epitope. EMBO.

[23] Stornaiuolo M, Lotti LV, Borgese N, Torrisi M-R, Mottola G, Martire G, Bonatti S (2003). KDEL and KKXX retrieval signals appended to the same reporter protein determine different trafficking between endoplasmic reticulum, intermediate compartment, and Golgi complex. Mol Biol Cell.

[24] Iqbal N, Iqbal N (2014). Human epidermal growth factor receptor 2 (HER2) in cancers: overexpression and therapeutic implications. Mol Biol Int.

[25] Sigismund S, Avanzato D, Lanzetti L (2018). Emerging functions of the EGFR in cancer. Mol Oncol.

[26] Orlova A, Magnusson M, Eriksson TL, Nilsson M, Larsson B, Höidén-Guthenberg I, Widström C (2006). Tumor imaging using a picomolar affinity HER2 binding affibody molecule. Cancer Res.

[27] Göstring L, Chew MT, Orlova A, Höidén-Guthenberg I, Wennborg A, Carlsson J, Frejd FY (2010). Quantification of internalization of EGFR-binding Affibody molecules: methodological aspects. Int J Oncol.

[28] Neubig RR, Spedding M, Kenakin T, Christopoulos A (2003). International Union of Pharmacology Committee on Receptor Nomenclature and Drug Classification. XXXVIII. Update on terms and symbols in quantitative pharmacology. Pharmacol Rev.

[29] Jia J, Yue X, Zhu L, Jing S, Wang Y, Gim B, Qian Y, Lee I (2021). KDEL receptor is a cell surface receptor that cycles between the plasma membrane and the Golgi via clathrin-mediated transport carriers. Cell Mol Life Sci.

[30] Becker B, Shaebani MR, Rammo D, Bubel T, Santen L, Schmitt MJ (2016). Cargo binding promotes KDEL receptor clustering at the mammalian cell surface. Sci Rep.

[31] Maxfield FR, McGraw TE (2004). Endocytic recycling. Nat Rev Mol Cell Biol.

[32] Johannes L, Popoff V (2008). Tracing the retrograde route in protein trafficking. Cell.

[33] Caliceti P, Veronese FM (2003). Pharmacokinetic and biodistribution properties of poly (ethylene glycol)–protein conjugates. Adv Drug Del Rev.

[34] Johansson MU, Frick I-M, Nilsson H, Kraulis PJ, Hober S, Jonasson P, Linhult M (2002). Structure, specificity, and mode of interaction for bacterial albumin-binding modules. J Biol Chem.

[35] Zaman R, Islam RA, Ibnat N, Othman I, Zaini A, Lee CY, Chowdhury EH (2019). Current strategies in extending half-lives of therapeutic proteins. J Control Release.

[36] Wei J, Bera TK, Liu XF, Zhou Q, Onda M, Ho M, Tai C-H, Pastan I (2018). Recombinant immunotoxins with albumin-binding domains have long half-lives and high antitumor activity. Proc Natl Acad Sci USA.

[37] Zakeri B, Fierer JO, Celik E, Chittock EC, Schwarz-Linek U, Moy VT, Howarth M (2012). Peptide tag forming a rapid covalent bond to a protein, through engineering a bacterial adhesin. Proc Natl Acad Sci USA.

[38] Thorpe PE, Wallace PM, Knowles PP, Relf MG, Brown AN, Watson GJ, Blakey DC, Newell DR (1988). Improved antitumor effects of immunotoxins prepared with deglycosylated ricin A-chain and hindered disulfide linkages. Cancer Res.

[39] Krolick K, Villemez C, Isakson P, Uhr J, Vitetta E (1980). Selective killing of normal or neoplastic B cells by antibodies coupled to the A chain of ricin. Proc Natl Acad Sci USA.

[40] Olsnes S (2004). The history of ricin, abrin and related toxins. Toxicon.

[41] Schnell R, Borchmann P, Staak J, Schindler J, Ghetie V, Vitetta E, Engert A (2003). Clinical evaluation of ricin A-chain immunotoxins in patients with Hodgkin’s lymphoma. Ann Oncol.

[42] Schnell R, Staak O, Borchmann P, Schwartz C, Matthey B, Hansen H, Schindler J (2002). A Phase I study with an anti-CD30 ricin A-chain immunotoxin (Ki-4. dgA) in patients with refractory CD30+ Hodgkin’s and non-Hodgkin’s lymphoma. Clin Cancer Res.

[43] Schnell R, Vitetta E, Schindler J, Borchmann P, Barth S, Ghetie V, Hell K (2000). Treatment of refractory Hodgkin’s lymphoma patients with an anti-CD25 ricin A-chain immunotoxin. Leukemia.

[44] Sausville EA, Headlee D, Stetler-Stevenson M, Jaffe ES, Solomon D, Figg WD, Herdt J (1995). Continuous infusion of the anti-CD22 immunotoxin IgG-RFB4-SMPT-dgA in patients with B-cell lymphoma: a phase I study. Blood.

[45] Schmidberger H, King L, Lasky LC, Vallera DA (1990). Antitumor activity of L6-ricin immunotoxin against the H2981–T3 lung adenocarcinoma cell line in vitro and in vivo. Cancer Res.

[46] Laske DW, Muraszko KM, Oldfield EH, DeVroom HL, Sung C, Dedrick RL, Simon TR (1997). Intraventricular immunotoxin therapy for leptomeningeal neoplasia. Neurosurgery.

[47] Lynch TJ, Lambert JM, Coral F, Shefner J, Wen P, Blattler WA, Collinson AR (1997). Immunotoxin therapy of small-cell lung cancer: a phase I study of N901-blocked ricin. J Clin Oncol.

[48] Tagge E, Chandler J, Tang BL, Hong W, Willingham MC, Frankel A (1996). Cytotoxicity of KDEL-terminated ricin toxins correlates with distribution of the KDEL receptor in the Golgi. J Histochem Cytochem.

[49] Zhan J, Stayton P, Press OW (1998). Modification of ricin A chain, by addition of endoplasmic reticulum (KDEL) or Golgi (YQRL) retention sequences, enhances its cytotoxicity and translocation. Cancer Immunol, Immunother.

[50] Ho M-C, Sturm MB, Almo SC, Schramm VL (2009). Transition state analogues in structures of ricin and saporin ribosome-inactivating proteins. Proc Natl Acad Sci USA.

