# Supplementary material for: Directing ricin-based immunotoxins with targeting affibodies and KDEL signal peptide to cancer cells effectively induces apoptosis and tumor suppression
Source: J Nanobiotechnology. 2022 Aug 23;20:387. doi: 10.1186/s12951-022-01601-8 (PMC9400252; doi:10.1186/s12951-022-01601-8)
Supplement: Supplementary file 1 — Additional file 1: Figure S1. In vitro quantitative measurements of dose-dependent cytotoxicity of HER2Afb-RTA or doxorubicin to (A) NIH3T6.7 and (B) SK-BR-3 cells and EGFRAfb-RTA, or doxorubicin to (C) MDA-MB-468 and (D) A431 cells. HER2Afb-RTA and EGFRAfb-RTA exhibited moderate or low cytotoxicity against target cells, compared to doxorubicin. All data shown are means ± standard deviation; n = 3. Curves are fitted using a Hill equation. Figure S2. Amino acid sequence information of (A) HER2Afb-RTA and HER2Afb-RTA-KDEL and (B) EGFRAfb-RTA and EGFRAfb-RTA-KDEL. The sequences of HER2Afb and EGFRAfb are indicated in blue and that of RTA is indicated in red. Linkers, his-tag, and KDEL signal peptide are indicated in black. (C) HER2Afb-RTA, HER2Afb-RTA-KDEL, EGFRAfb-RTA, EGFRAfb-RTA-KDEL, and RTA-KDEL were purified with IMAC and analyzed by SDS-PAGE. Figure S3. Fluorescence microscopic images of NIH3T6.7 cells treated with (A) HER2Afb-RTA or (C) HER2Afb-RTA-KDEL at various times. Nuclei, RTA, ER, and lysosomes are shown in blue, green, red, and white, respectively. Scale bars, 20 μm. One of cells in (A) and (C) was selected and fluorescent intensity profiles of (B) HER2Afb-RTA and (D) HER2Afb-RTA-KDEL in the selected area were analyzed. The fluorescent intensity of RTA is represented by colorimetric scale bar, and 2D projection of the area was drawn at the top of the 3D plot. (E) Fluorescent signal intensity of HER2Afb-RTA or HER2Afb-RTA-KDEL bound to the cell surface at 0 h was statistically measured by line intensity analysis and plotted as a bar graph. Data shown in the bar graph are the means ± SD; ****p < 0.0001. Figure S4. Fluorescence microscopic images of MDA-MB-468 cells treated with (A) EGFRAfb-RTA or (C) EGFRAfb-RTA-KDEL at various times. Nuclei, RTA, ER, and lysosomes are shown in blue, green, red, and white, respectively. Scale bars, 20 μm. One of cells in (A) and (C) was selected and fluorescent intensity profiles of (B) EGFRAfb-RTA and (D) EGFRAfb-RTA-KDE [file 12951_2022_1601_MOESM1_ESM.docx]

*Supporting information for*

**Directing Ricin-based Immunotoxins with Targeting Affibodies and KDEL Signal Peptide to Cancer Cells Effectively Induces Apoptosis and Tumor Suppression**

Seong Guk Park,^1,#^ Heeyeon Kim,^1,#^ Heejin Jun,^1^ Sun Young Choi,^2.^* Eunhee Kim,^1,^* and Sebyung Kang^1,^*

^1^Department of Biological Sciences, Ulsan National Institute of Science and Technology (UNIST), Ulsan, Korea and ^2^Department of Medicine, Graduate School, Korea University, Seoul, Korea

^#^These authors contributed equally to this work

Corresponding authors

Sebyung Kang: [sabsab7@unist.ac.kr](mailto:sabsab7@unist.ac.kr)

Eunhee Kim: [ehkim@unist.ac.kr](mailto:ehkim@unist.ac.kr)

Sun Young Choi: [csy4ycy2@gmail.com](mailto:csy4ycy2@gmail.com)


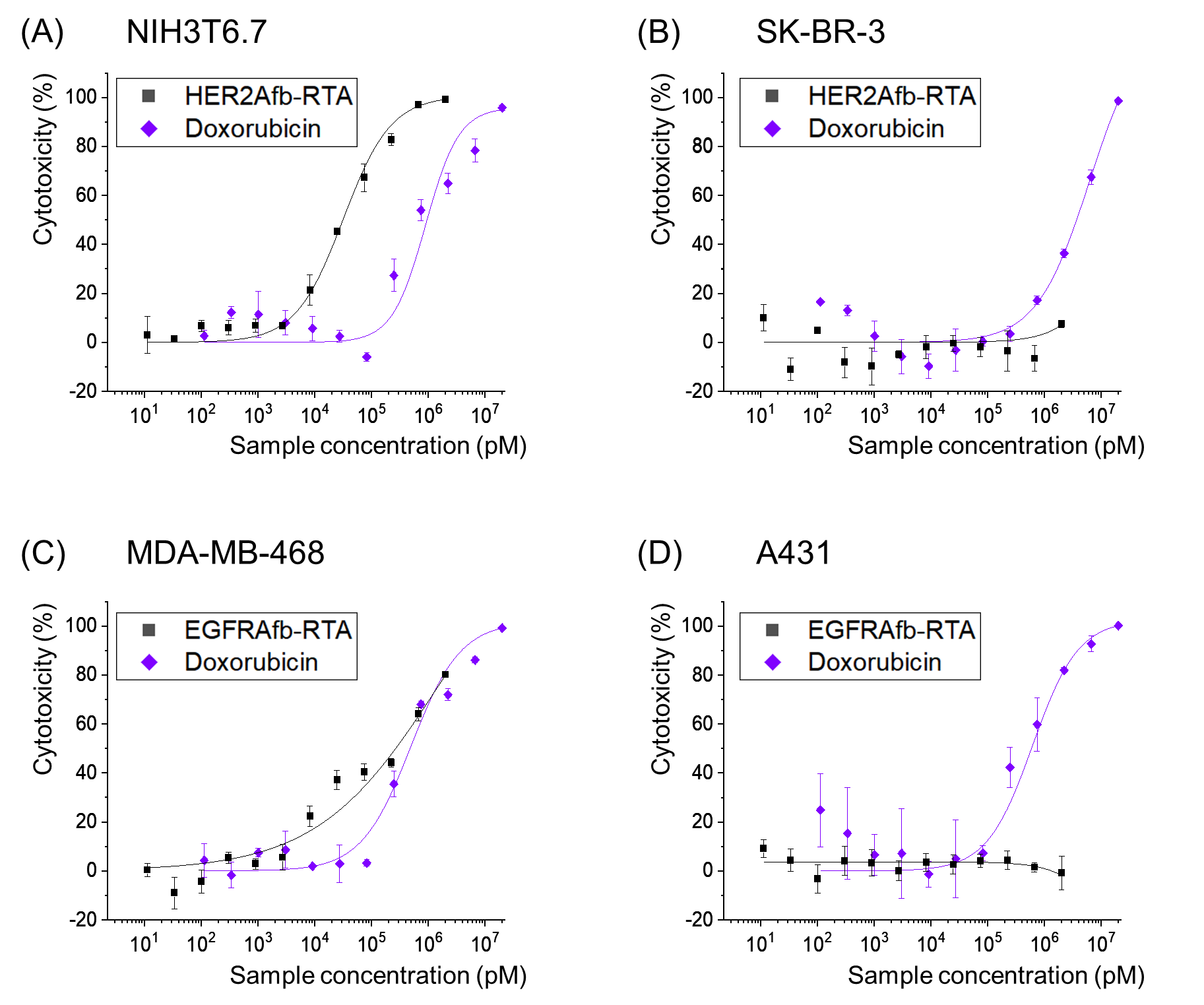


Figure S1. In vitro quantitative measurements of dose-dependent cytotoxicity of HER2Afb-RTA or doxorubicin to (A) NIH3T6.7 and (B) SK-BR-3 cells and EGFRAfb-RTA, or doxorubicin to (C) MDA-MB-468 and (D) A431 cells. HER2Afb-RTA and EGFRAfb-RTA exhibited moderate or low cytotoxicity against target cells, compared to doxorubicin. All data shown are means ± standard deviation; n=3. Curves are fitted using a Hill equation.


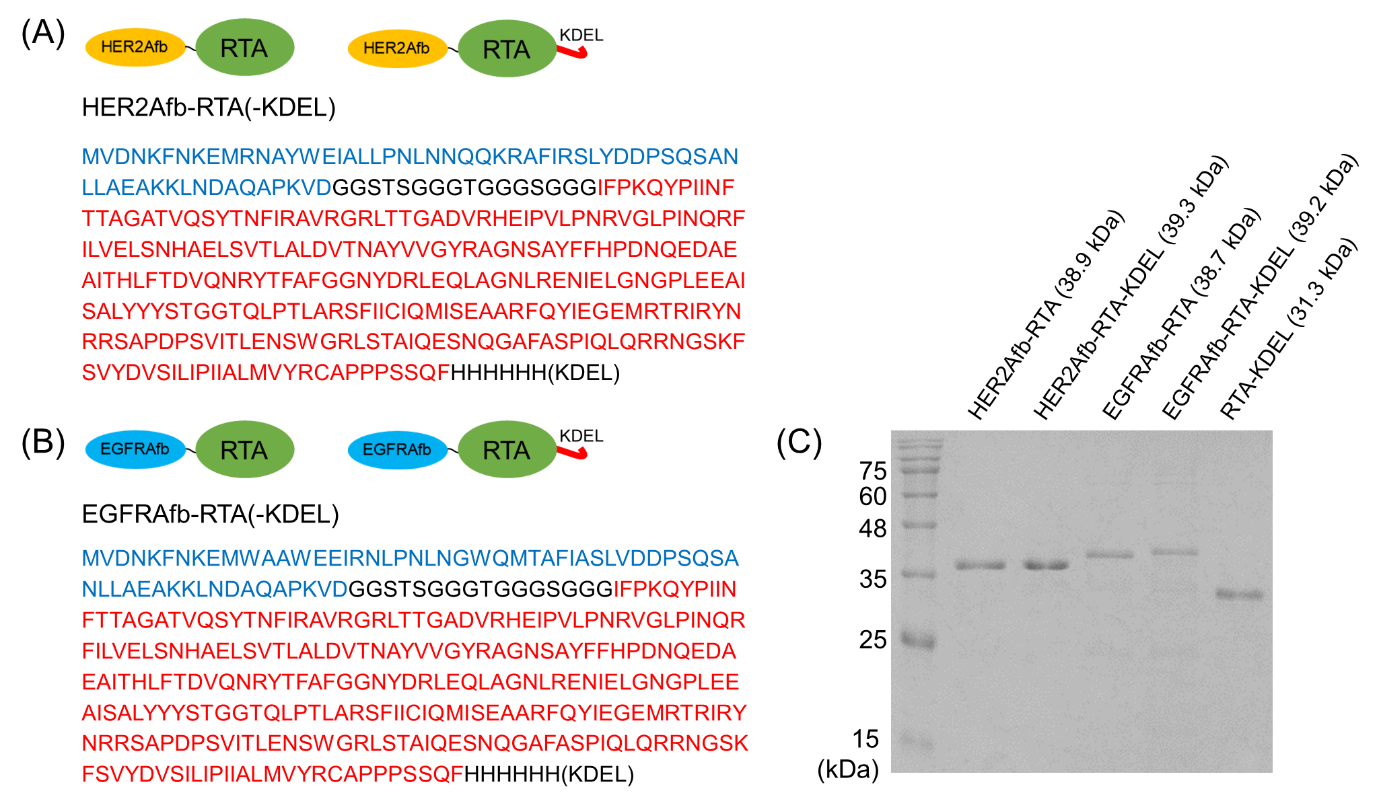


Figure S2. Amino acid sequence information of (A) HER2Afb-RTA and HER2Afb-RTA-KDEL and (B) EGFRAfb-RTA and EGFRAfb-RTA-KDEL. The sequences of HER2Afb and EGFRAfb are indicated in blue and that of RTA is indicated in red. Linkers, his-tag, and KDEL signal peptide are indicated in black. (C) HER2Afb-RTA, HER2Afb-RTA-KDEL, EGFRAfb-RTA, EGFRAfb-RTA-KDEL, and RTA-KDEL were purified with IMAC and analyzed by SDS-PAGE.


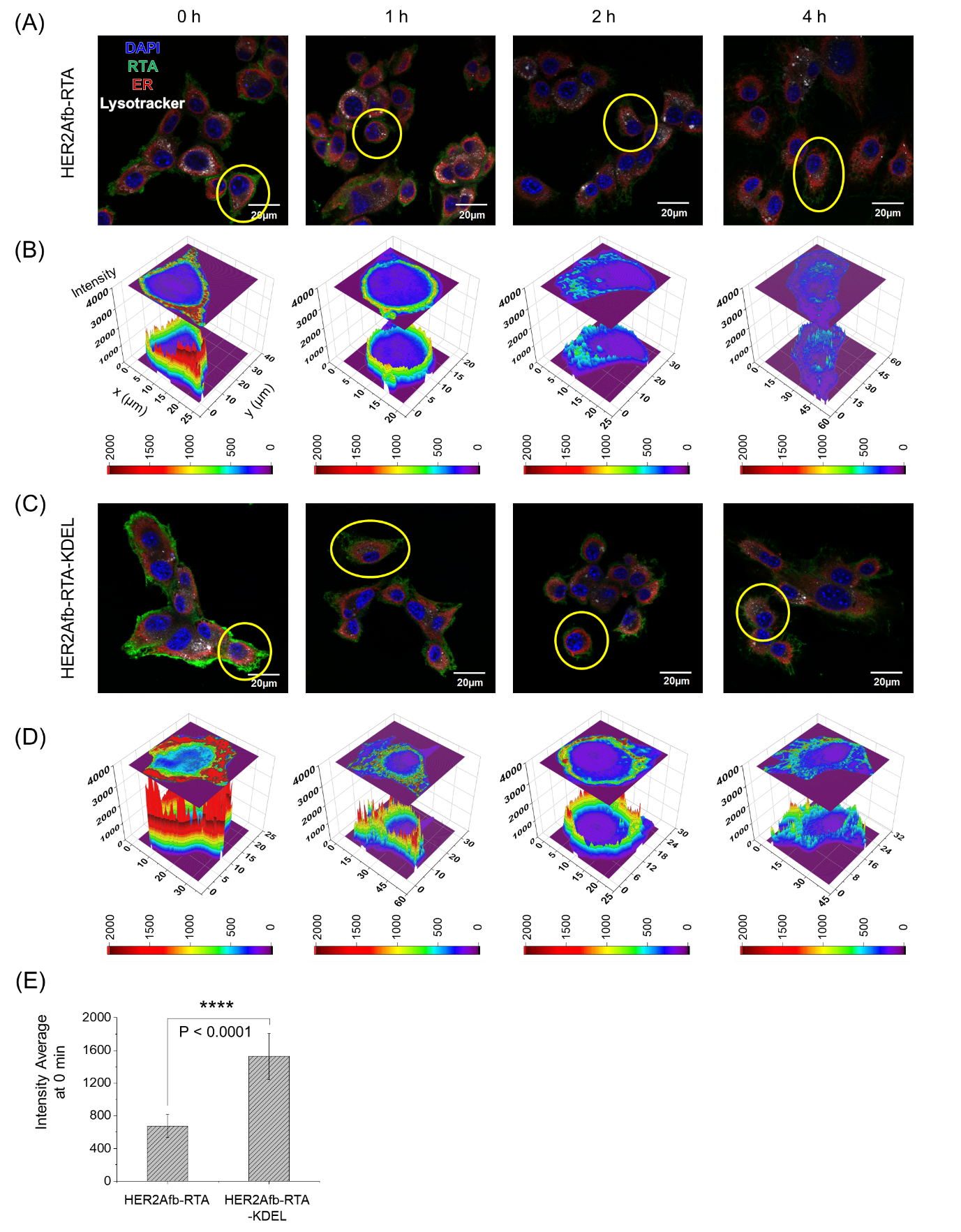


Figure S3. Fluorescence microscopic images of NIH3T6.7 cells treated with (A) HER2Afb-RTA or (C) HER2Afb-RTA-KDEL at various times. Nuclei, RTA, ER, and lysosomes are shown in blue, green, red, and white, respectively. Scale bars, 20 μm. One of cells in (A) and (C) was selected and fluorescent intensity profiles of (B) HER2Afb-RTA and (D) HER2Afb-RTA-KDEL in the selected area were analyzed. The fluorescent intensity of RTA is represented by colorimetric scale bar, and 2D projection of the area was drawn at the top of the 3D plot. (E) Fluorescent signal intensity of HER2Afb-RTA or HER2Afb-RTA-KDEL bound to the cell surface at 0 h was statistically measured by line intensity analysis and plotted as a bar graph. Data shown in the bar graph are the means ± SD; *****p* < 0.0001.


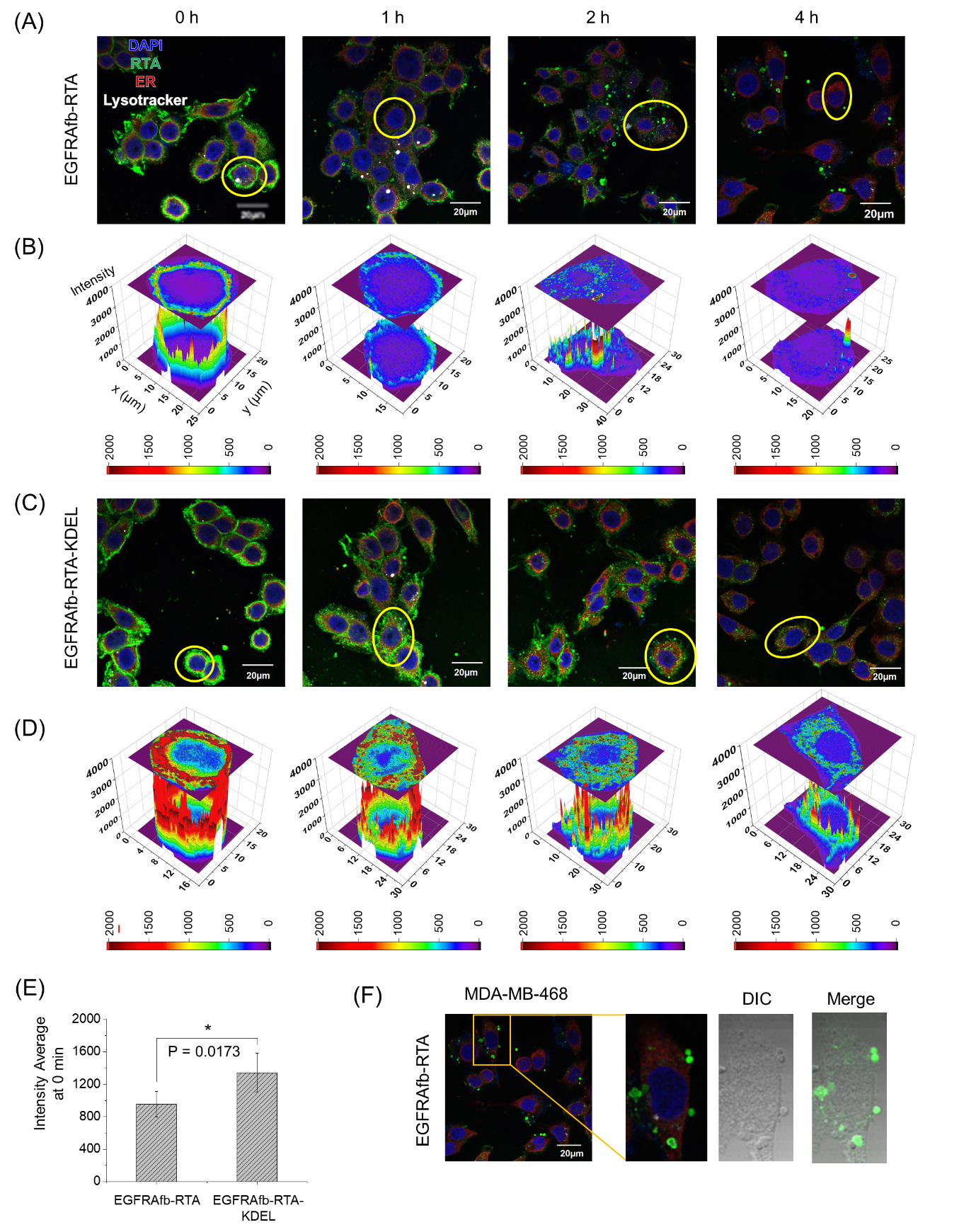


Figure S4. Fluorescence microscopic images of MDA-MB-468 cells treated with (A) EGFRAfb-RTA or (C) EGFRAfb-RTA-KDEL at various times. Nuclei, RTA, ER, and lysosomes are shown in blue, green, red, and white, respectively. Scale bars, 20 μm. One of cells in (A) and (C) was selected and fluorescent intensity profiles of (B) EGFRAfb-RTA and (D) EGFRAfb-RTA-KDEL in the selected area were analyzed. The fluorescent intensity of RTA is represented by colorimetric scale bar, and 2D projection of the area was drawn at the top of the 3D plot. (E) Fluorescent signal intensity of EGFRAfb-RTA or EGFRAfb-RTA-KDEL bound to the cell surface at 0 h was statistically measured by line intensity analysis and plotted as a bar graph. (F) Microvesicle-like structures containing RTA at the surface of MDA-MB-468 treated with EGFRAfb-RTA were enlarged with DIC images. Data shown in the bar graph are the means ± SD; **p* < 0.05


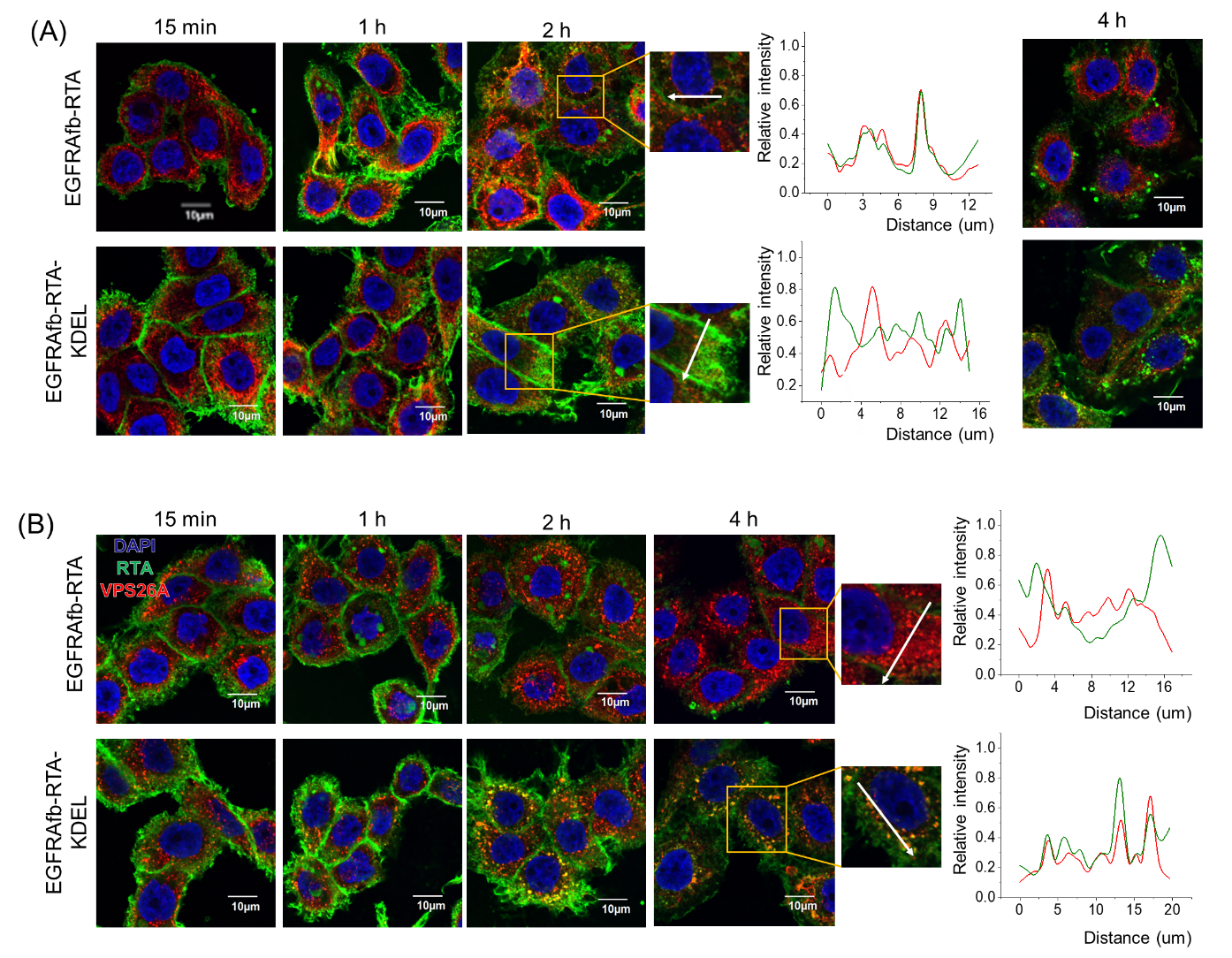
Figure S5. Fluorescence microscopic images of MDA-MB-468 cells treated with EGFRAfb-RTA (A, C) or EGFRAfb-RTA-KDEL (B, D) at various times. (A, B) Nuclei, RTA, and TfR were shown in blue, green, and red, respectively. (C, D) Nuclei, RTA, and VPS26A were shown in blue, green, and red, respectively. Co-localization of RTA and TfR (A, B at 2 h) or RTA and VPS26A (C, D at 4 h) was also analyzed with a line intensity profile according to distance. The analyzed lines are indicated as white arrows. Scale bars, 10 μm.


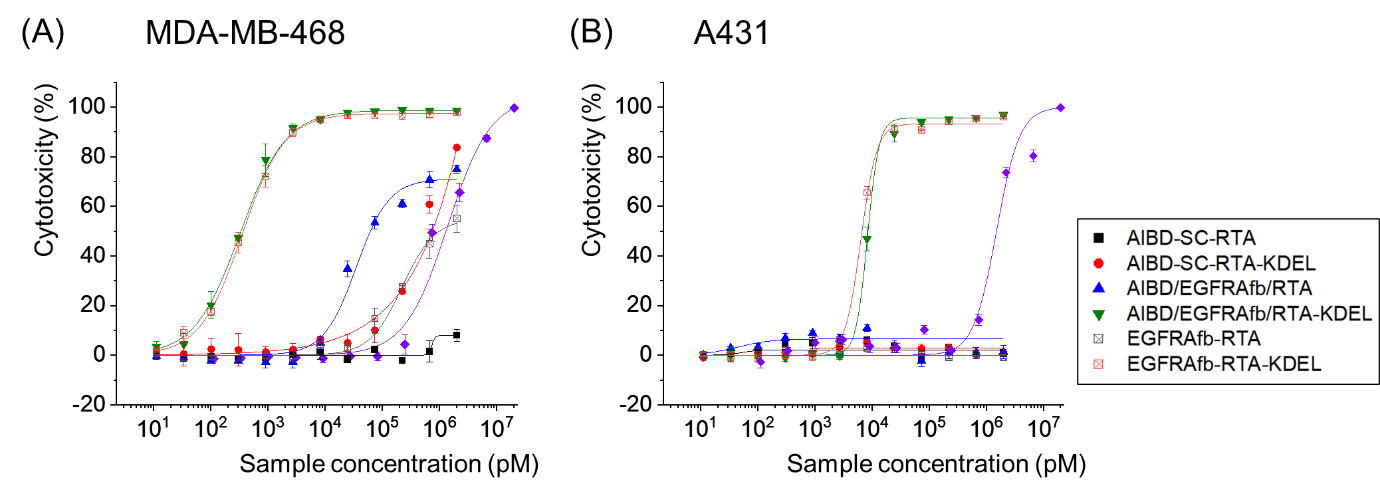


Figure S6. In vitro quantitative measurements of dose-dependent cytotoxicity of RTA variants to (A) MDA-MB-468 and (D) A431 cells with CCK-8 assay. All data shown are means ± standard deviation; n=3. Curves are fitted using a Hill equation.


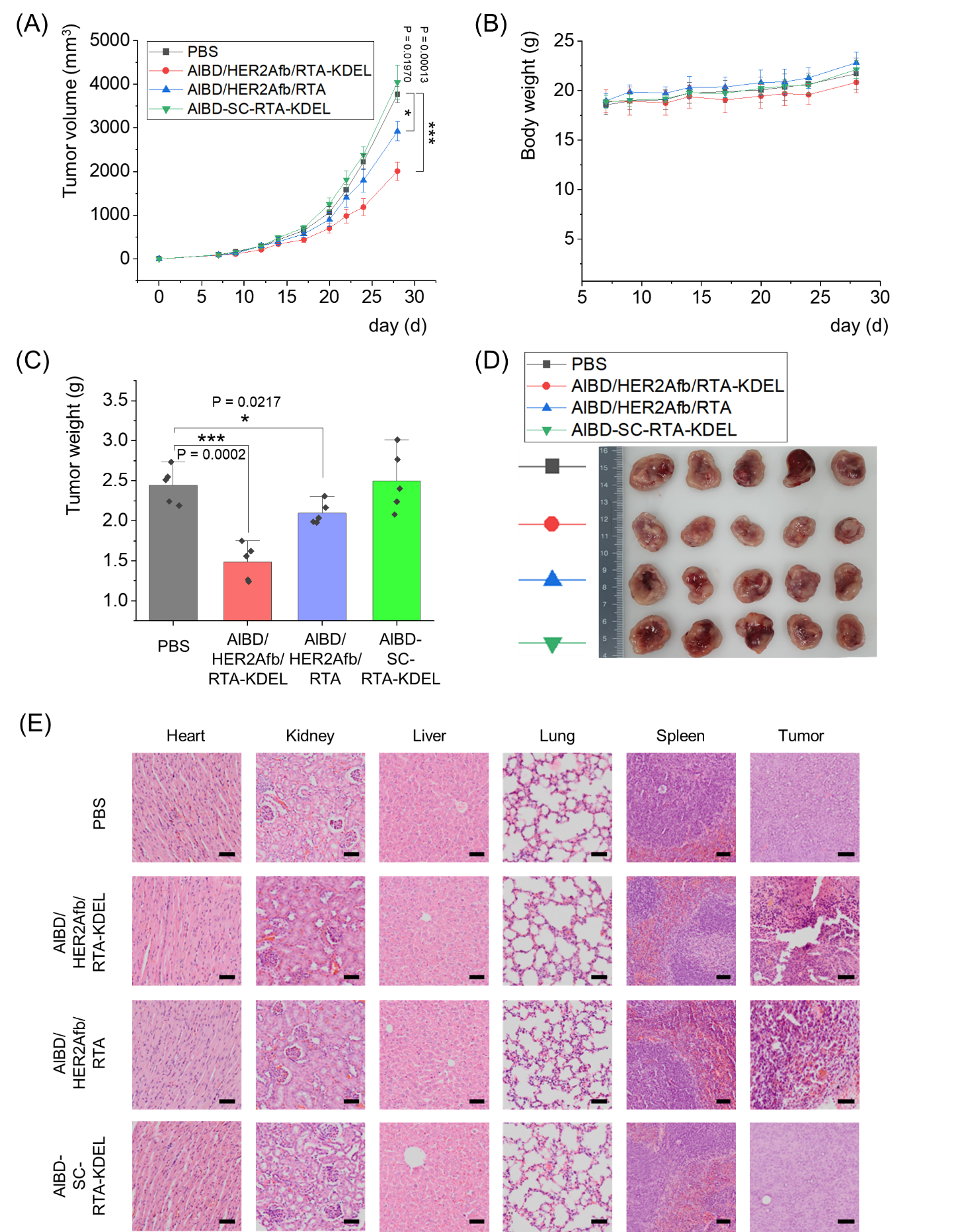


Figure S7. In vivo therapeutic efficacy of AlBD/HER2Afb/RTA-KDEL and other RTA variants against NIH3T6.7 tumor-bearing mice. NIH3T6.7 cells were allografted to nude mice on day 0. After the tumor volume reached approximately 100 mm^3^, PBS, AlBD/HER2Afb/RTA-KDEL, AlBD/HER2Afb/RTA, and AlBD-SC-RTA-KDEL (5 μg/100 μl each) were intravenously injected for a total of eight times in 2 or 3 day intervals. (A) tumor volumes and (B) body weights were measured with a caliper and scaler, respectively. (C) Weight of biopsied tumors were measured and (D) picture of the biopsied tumors are shown. (E) The hearts, kidneys, livers, lungs, spleens, and tumors collected from sacrificed mice treated with PBS, AlBD/HER2Afb/RTA-KDEL, AlBD/HER2Afb/RTA, and AlBD-SC-RTA-KDEL were fixed, embedded in paraffin, and sectioned. The organ tissue and tumor sections were stained with H&E. Images were captured using an Olympus virtual microscope. Scale bar, 50 μm. All data shown in (A-C) are the means ± SD; n = 5 per group; **p* < 0.05; ***p* < 0.01; ****p* < 0.001
